# An Evolutionarily Conserved Mechanism for Intrinsic and Transferable Polymyxin Resistance

**DOI:** 10.1128/mBio.02317-17

**Published:** 2018-04-10

**Authors:** Yongchang Xu, Wenhui Wei, Sheng Lei, Jingxia Lin, Swaminath Srinivas, Youjun Feng

**Affiliations:** a Department of Medical Microbiology and Parasitology, Zhejiang University School of Medicine, Hangzhou, Zhejiang, China; b College of Animal Sciences, Zhejiang University, Hangzhou, Zhejiang, China; c Department of Biochemistry, University of Illinois at Urbana-Champaign, Urbana, Illinois, USA; University of Siena; Indiana University Bloomington

**Keywords:** enteric bacteria, EptA, lipid A, MCR-1, MCR-2, polymyxin resistance, substrate cavity

## Abstract

Polymyxins, a family of cationic antimicrobial cyclic peptides, act as a last line of defense against severe infections by Gram-negative pathogens with carbapenem resistance. In addition to the intrinsic resistance to polymyxin E (colistin) conferred by *Neisseria eptA*, the plasmid-borne mobilized colistin resistance gene *mcr-1* has been disseminated globally since the first discovery in Southern China, in late 2015. However, the molecular mechanisms for both intrinsic and transferable resistance to colistin remain largely unknown. Here, we aim to address this gap in the knowledge of these proteins. Structural and functional analyses of EptA and MCR-1 and -2 have defined a conserved 12-residue cavity that is required for the entry of the lipid substrate, phosphatidylethanolamine (PE). The *in vitro* and *in vivo* data together have allowed us to visualize the similarities in catalytic activity shared by EptA and MCR-1 and -2. The expression of either EptA or MCR-1 or -2 is shown to remodel the surface of enteric bacteria (e.g., Escherichia coli, Salmonella enterica, Klebsiella pneumoniae, etc.), rendering them resistant to colistin. The parallels in the PE substrate-binding cavities among EptA, MCR-1, and MCR-2 provide a comprehensive understanding of both intrinsic and transferable colistin resistance. Domain swapping between EptA and MCR-1 and -2 reveals that the two domains (transmembrane [TM] region and phosphoethanolamine [PEA] transferase) are not functionally exchangeable. Taken together, the results represent a common mechanism for intrinsic and transferable PEA resistance to polymyxin, a last-resort antibiotic against multidrug-resistant pathogens.

## IMPORTANCE

EptA and MCR-1 and -2 remodel the outer membrane, rendering bacteria resistant to colistin, a final resort against carbapenem-resistant pathogens. Structural and functional analyses of EptA and MCR-1 and -2 reveal parallel PE lipid substrate-recognizing cavities, which explains intrinsic and transferable colistin resistance in gut bacteria. A similar mechanism is proposed for the catalytic activities of EptA and MCR-1 and -2. Together, they constitute a common mechanism for intrinsic and transferable polymyxin resistance.

## INTRODUCTION

**FIG 1:**
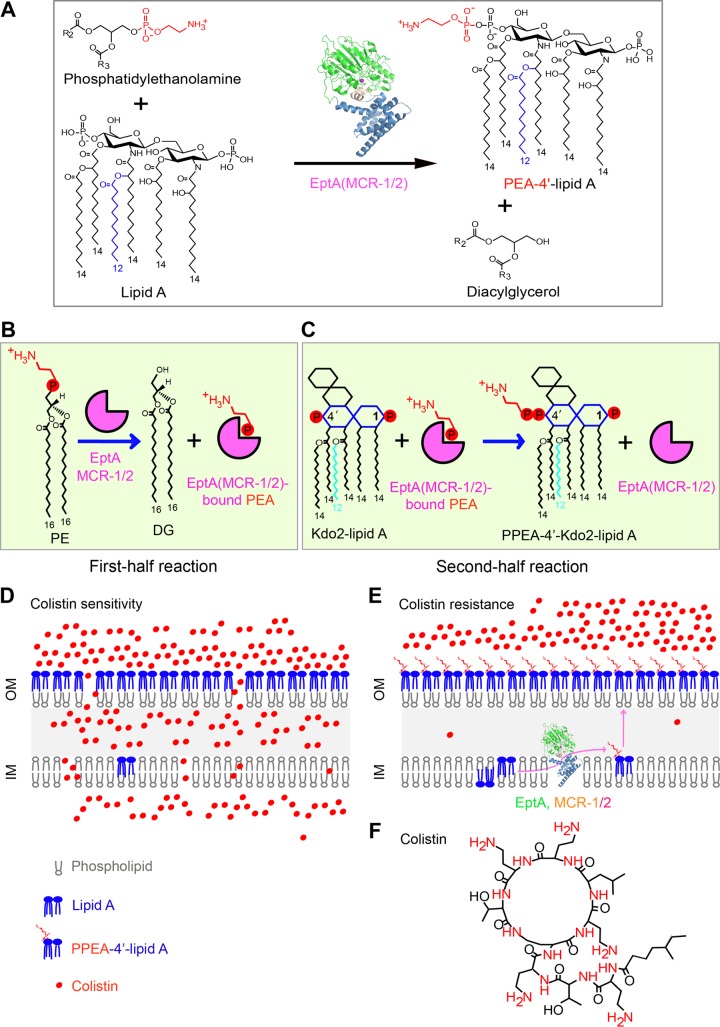
Schemes of chemical mechanism and colistin resistance of MCR-1, MCR-2, and EptA. (A) Chemical reaction for the transfer of PEA to lipid A by MCR-2. MCR-2 catalyzes the addition of PEA to position 1 or 4′ of lipid A, giving the final products PEA-1(4′)-lipid A and diacylglycerol. The enzyme (MCR-1 or -2 or EptA) is depicted with a ribbon structure comprising the N-terminal catalytic domain (in green) and the transmembrane domain (in blue) at the C terminus. The chemical structures of molecules are illustrated with ChemDraw software. (B) The first half reaction of MCR-1 or -2 or EptA is defined by the removal of PEA from PE, giving the final product DG and the intermediate product enzyme-bound PEA. (C) The second half reaction of MCR-1 or -2 or EptA comprises the generation of the final product Kdo2 [di(3-deoxy-d-manno-octulosonic acid)]-lipid A-4′-PPEA through the transfer of PEA from the adduct of enzyme-bound PEA to the recipient Kdo2-lipid A. (D) Cartoon of the model proposed for the bacterial surface structure of colistin-sensitive E. coli. (E) Working model for structural modification of lipid A anchored on bacterial surface involved in colistin resistance. PE, phosphatidylethanolamine; DG, diacylglycerol; PEA, phosphoethanolamine; OM, outer membrane; IM, inner membrane. (F) Chemical structure of the cationic antibiotic peptide, colistin. Positively charged elements are denoted in red.

The growing antibiotic resistance emerging in microbial pathogens is a leading challenge to global public health (1–3). The rapid evolution of multidrug-resistant (MDR) organisms has almost pushed us to the cusp of a postantibiotic era. Data from the U.S. CDC (Centers for Disease Control and Prevention) indicate that more than 700,000 deaths worldwide occur every year due to infections caused by MDR pathogens (1, 2), with about 23,000 of those being recorded in the United States alone (4). The emergence of New Delhi β-lactamase 1 (NDM-1) and/or its variants, in addition to the Klebsiella pneumoniae carbapenemase (KPC), has called into question the use of carbapenem, a β-lactam antibiotic, as an effective treatment option against severe MDR infections (5, 6). Consequently, the cationic antimicrobial peptide polymyxin (regardless of its partial renal toxicity) was reintroduced as a last-resort option for the clinical treatment of patients infected by the carbapenem-resistant Gram-negative bacterium (7). However, it seems that that bacteria have developed either an acquired resistance to colistin or a chromosomally encoded “intrinsic” resistance (Fig. 1) (8). Intrinsic resistance is naturally occurring and is due to the functional expression of certain chromosomal genes (such as *eptA* of Neisseria meningitidis). Acquired resistance to colistin is evidently related to mutations in two-component regulatory systems involved in the lipopolysaccharide synthesis pathway (e.g., PhoP/PhoQ in K. pneumoniae [9] and Pseudomonas aeruginosa [10] and PmrA/PmrB in Salmonella enterica [11–13], Acinetobacter baumannii [14], and P. aeruginosa [15]). Transferable resistance to colistin is conferred/acquired through plasmid-borne genes (i.e., *mcr-1* and *mcr-2*) (16, 17). Therefore, new strategies are urgently required to reverse/bypass colistin resistance in Gram-negative pathogens (18).

Colistin primarily interacts with the lipopolysaccharide (LPS) layer of the Gram-negative bacterial outer membrane (19–21). A structural alteration of LPS species that reduces the overall negative charge of the bacterial surface and interferes with the electrostatic interaction between colistin and LPS (22) renders the bacteria resistant to colistin (Fig. 1A) (17). Of all the covalent modifications of LPS that occur, the addition of phosphoethanolamine (PEA [also called pEtN]) to lipid A is the most prevalent (12, 23). EptA in *Neisseria*, a member of the PEA transferase family, is a well-known player in catalyzing the transfer of PEA to the 1-phosphate or 4′-phosphate [1(4′)-phosphate] position of lipid A glucosamine (GlcN) moieties (22, 24). However, unlike the chromosomal *eptA* (22), *mcr-1*, a newly identified gene encoding a PEA transferase, is harbored on diversified plasmids that disseminate into a variety of *Enterobacteriaceae* species and confers a transferable resistance to colistin (25, 26). In fact, *mcr-1* has spread to over 40 countries or regions, covering 5 of the 7 continents (27, 28), and is the most-frequently detected genetic element of mobilized colistin resistance, especially on swine farms with massive use of colistin. Very recently, MCR-2 was determined to be a new variant of MCR-1, with around 80% identity and exclusively encoded on an IncX4-type plasmid (16, 17). This seems to be a rare form in that it has only been detected in Belgium (29, 30). In addition, three more *mcr*-like genes have been elucidated (28), namely, *mcr-3* (31–35), *mcr-4* (36), and *mcr-5* (37). The MCR-like enzymes encoded by these genes exhibit relatively low levels of similarity to MCR-1 and -2 (28). Together with other research groups (38–42), we have defined a zinc-requiring catalytic motif in MCR-1 that is a prerequisite for colistin resistance (43, 44). In addition, we have also deciphered the partially genetic/biochemical mechanism of MCR-2 for colistin resistance (17). However, we are not aware of how MCR-2 interacts with the phosphatidylethanolamine (PE) substrate, due to a lack of structural/chemical evidence.

In this study, we report mechanistic insights into the structure and function of the two PEA transferases EptA and MCR-2. In addition to the overall architecture, we also illustrate a parallel between their substrate entry cavities. More importantly, we propose that a possible “ping-pong” mechanism is shared among EptA, MCR-1, and MCR-2 (Fig. 1B and C). This mechanism features the PEA transfer via the formation of an intermediate product, enzyme-bound PEA. Our finding reveals that parallels are present between intrinsic and transferable resistance to colistin (Fig. 1D to F), a last-resort treatment option for severe infections by carbapenem-resistant superbugs.

## RESULTS

### Discovery of a cavity for PE substrate entry in EptA.

**FIG 2:**
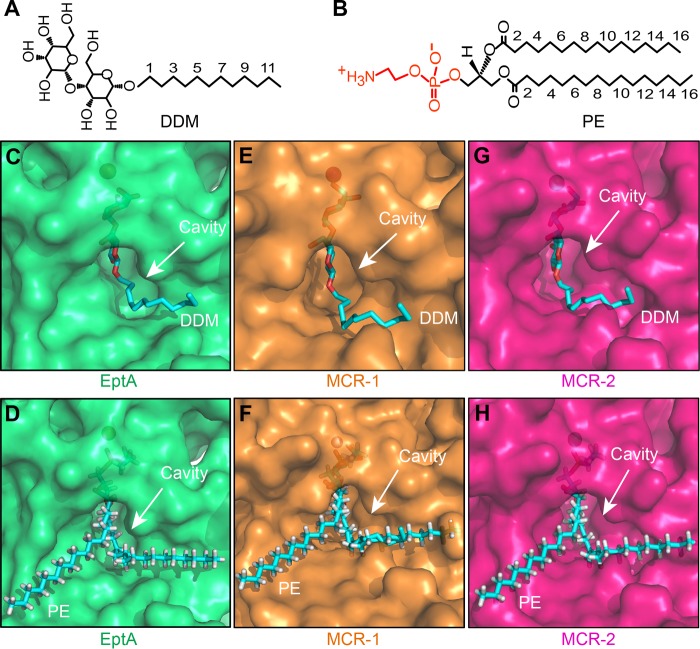
Identification of a conserved PE substrate-binding cavity among EptA and MCR-1 and -2. (A) Chemical structure of the detergent DDM, an analogue of the PE lipid substrate for MCR-1 and -2. (B) Chemical structure of the PE molecule, the lipid substrate of MCR-1 and -2. (C to H) Surface structure illustrations of the DDM-bound cavities in EptA (C), MCR-1 (E), and MCR-2 (G) and comparison of surface structures of the PE-bound cavities in EptA (D), MCR-1 (F), and MCR-2 (H). DDM/PE molecules are illustrated with blue sticks, and cavity is highlighted with an arrow. Images were created using PyMol. DDM, *n*-dodecyl-β-d-maltoside; PE, phosphatidyl ethanolamine.

The *Neisseria* EptA (LptA [lipid A PEA transferase]), which belongs to the YhjW/YjdB/YijP superfamily, is a previously known intrinsic determinant of colistin resistance. The X-ray structure of full-length EptA revealed a previously uncharacterized helical membrane domain (see Fig. S1 and S2 in the supplemental material) (24), suggesting residues that might be involved in PE substrate binding (45). However, experimental evidence for this is still missing. We therefore applied molecular docking to reanalyze the complex structure of EptA with a PE substrate analogue, the detergent *n*-dodecyl β-d-maltoside (DDM) (Fig. 2A and B). As a result, we propose a cavity (Fig. 2B and D), 12 residues of which are essential for PE substrate entry/binding (Fig. S2A to D). Among them, 5 amino acids (E240, T280, H378, D452, and H453) are involved in the interaction with zinc (Fig. S1, S2B, and S2F) and the remaining 7 residues (N106, T110, E114, S325, K328, H383, and D465) are implicated in recognition of the physiological substrate, PE (Fig. S2D and G). To elucidate the function of these residues that define the cavity, we generated 12 point mutants of EptA and demonstrated their effectiveness using colistin resistance assays. Even though all EptA point mutants are expressed well in Escherichia coli, as verified by Western blotting (Fig. S2E), none of the five mutants with point mutations of EptA with an alanine substitution in a zinc-binding site can support any significant growth in the presence of colistin (Fig. S2F). As for the point mutants with mutations of the 7 PE substrate-interacting sites, 3 are partially inactive in the trials of colistin resistance on Luria-Bertani agar (LBA) plates, namely, those with the N106A (bearing a change of N to A at position 106) (4 μg/ml), S325A (2 μg/ml), and T110A (1 μg/ml) mutations (Fig. S2G), while the remaining four mutants are fully nonfunctional, i.e., their mutations confer no additional resistance to colistin, in agreement with the results for the negative control (0.5 μg/ml) (Fig. S2G). Similar scenarios were also observed in the colistin MIC measurements (Fig. S2H). The aforementioned *in vivo* data thus confirm the importance of cavity-forming residues in the ability of EptA to provide resistance to colistin.

10.1128/mBio.02317-17.1 FIG S1  Sequence alignment of three colistin resistance genes MCR-1, MCR-2, and NgEptA. The transmembrane region, underlined in blue, is revealed through prediction with TMHMM server version 2.0 (http://www.cbs.dtu.dk/services/TMHMM/). The multiple sequence alignment was carried out using Clustal Omega (https://www.ebi.ac.uk/Tools/msa/clustalo/), with the current output by the program ESPript 3.0 (http://espript.ibcp.fr/ESPript/cgi-bin/ESPript.cgi) (61). The five Zn^2+^-interacting residues are highlighted with red arrows, and the seven PE substrate-binding amino acids are indicated with blue arrows. Identical residues are in white letters with red background, similar residues are in red letters with white background, and varied residues are in black letters. The protein secondary structure is illustrated in cartoon form (on top). α, α-helix; β, beta-sheet; T, turn; η, coil; TMH, transmembrane helix; PH, periplasmic-facing helix; Ng, Neisseria gonorrhoeae. Download FIG S1, JPG file, 0.6 MB. Copyright © 2018 Xu et al. 2018 Xu et al. This content is distributed under the terms of the Creative Commons Attribution 4.0 International license.

10.1128/mBio.02317-17.2 FIG S2  Structural reanalysis of *Neisseria gonorrhea* EptA defines a functional cavity for the entry and binding of PE substrate molecules. (A) Schematic illustration of surface structure of *N. gonorrhea* EptA (NgEptA). (B) Enlarged view of a 5-residue motif implicated in Zn^2+^ binding. (C) Surface structure of NgEptA rotated 35°C in the reverse direction. (D) Enlarged view of a 7-residue motif having a role in the PE substrate-binding cavity of NgEptA. The 7 amino acids involved into the PE substrate-binding cavity of NgEptA are N106, T110, E114, S325, K328, H383, and H465. (E) Western blot analyses of the point mutants of EptA with anti-6×His rabbit antiserum as primary antibody. (F) Functional mapping of the Zn^2+^-binding motif using assays of colistin resistance on LBA plates. The five residues (E240, T280, H378, D452, and H453) are required for Zn^2+^ binding of NgEptA. (G) Site-directed mutagenesis-based analyses of the PE-binding cavity in the context of colistin resistance conferred by NgEptA. The two periplasmic-facing helices (in light pink) participate in the binding of NgEptA to PE substrate molecules in that they carry the three essential amino acids (namely, N106, T110, and E114). (H) Comparative analyses of colistin MICs in the E. coli strains carrying either the wild-type NgEptA or its point mutants. Levels of colistin resistance were assayed using LBA plates (F and G). A representative result from no less than three independent trials is given. Vec, empty pBAD24 vector. Colistin MIC trials were conducted using the micro-broth dilution method, and the breakpoint was set according to the guidelines of the European Committee on Antimicrobial Susceptibility Testing (EUCAST 2015, version 5.0) (70). Ng, Neisseria gonorrhoeae. Download FIG S2, JPG file, 0.5 MB. Copyright © 2018 Xu et al. 2018 Xu et al. This content is distributed under the terms of the Creative Commons Attribution 4.0 International license.

As described by Anandan et al. (45), we also assessed the role of this cavity in the enzymatic activity of EptA *in vitro*, and found it to be similar to that of MCR-2 (see Fig. 4). As anticipated, the results are in agreement with those of our colistin trials (Fig. S2) and show that (i) each of the 5 alanine mutants of EptA with defects in zinc binding have no detectable activity and (ii) 3 of 7 EptA derivatives with mutations in the PE-recognizing cavity (N106A, T110A, and S325A) possess partial activity, whereas the other 4 (E114A, K328A, H383A, and D465A) are inactive (data not shown). Subsequently, we subjected the lipid A of E. coli cells expressing *eptA* mutants to matrix-assisted laser desorption ionization–time of flight mass spectrometry (MALDI-TOF MS) (Table 1). We observed that EptA can modify lipid A (mass-to-charge ratio [*m/z*], 1,796.700 to 1,797.936) to phosphoethanolamine-1(4′)-lipid A [PPEA-1(4′)-lipid A] (*m/z*, 1,919.983) (Table 1). Second, 3 of 12 EptA mutants (N106A, T110A, and S325A) still retain partial activity in transferring the PEA moiety to lipid A, as evidenced by an additional appearance of PPEA-1(4′)-lipid A (*m/z*, 1,920.077 to 1,920.195) (Table 1). Third, the remaining 9 mutants of EptA are nonfunctional in catalyzing the formation of PPEA-1(4′)-lipid A, since the MS peak of lipid A alone is consistently at an *m/z* of 1,796.719 to 1,797.069 (Table 1). In addition to the three mutants (E114A, E240A, and T280A) with partial activity (Table 1), six more derivatives of EptA (K328A, H378A, H383A, D452A, H453A, and H456A mutants) are involved in the enzyme activity (Table 1). These results constitute a functional definition of a cavity in EptA for PE lipid substrate entry.

**TABLE 1:** MALDI-TOF MS profiles of lipid A species from E. coli MG1655 strains expressing *eptA*, *mcr-1*, *mcr-2*, and their point mutants

Protein, lipid species	Mass (*m/z*) of lipid species from *E. coli* MG1655 bearing:													
	Empty vector	No vector (WT^a^)	Vector expressing protein with indicated mutation											
EptA			N106A	T110A	E114A	E240A	T280A	S325A	K328A	H378A	H383A	D452A	H453A	H465A
Lipid A	1,796.700	1,797.936	1,797.02	1,797.083	1,796.950	1,797.069	1,796.719	1,797.138	1,797.005	1,796.893	1,796.734	1,796.935	1,796.814	1,796.869
PPEA-4′-lipid A		1,919.983	1,920.077	1,920.141				1,920.195						
MCR-1^b^			N108A	T112A	E116A	E246A	T285A	S330A	K333A	H390A	H395A	D465A	H466A	H478A
Lipid A	1,796.567	1,796.915	1,796.572	1,796.707	1,796.598	1,796.645	1,796.521	1,796.651	1,796.676	1,796.786	1,796.535	1,796.675	1,796.718	1,796.912
PPEA-4′-lipid A		1,919.969	1,919.614	1,919.753				1,919.695						
MCR-2			N106A	T110A	E114A	E244A	T283A	S328A	K331A	H388A	H393A	D463A	H464A	H476A
Lipid A	1,796.243	1,796.939	1,796.986	1,796.812	1,796.622	1,796.571	1,796.722	1,796.717	1,796.538	1,796.860	1,796.070	1,796.611	1,796.602	1,796.664
PPEA-4′-lipid A		1,919.991	1,919.114	1,919.857				1,919.753						

a WT, wild type.

b The mass data for MCR-1 are adapted from the recent description by our research group (44).

### Similarity among PE-binding cavities of EptA, MCR-1, and MCR-2.

**FIG 3:**
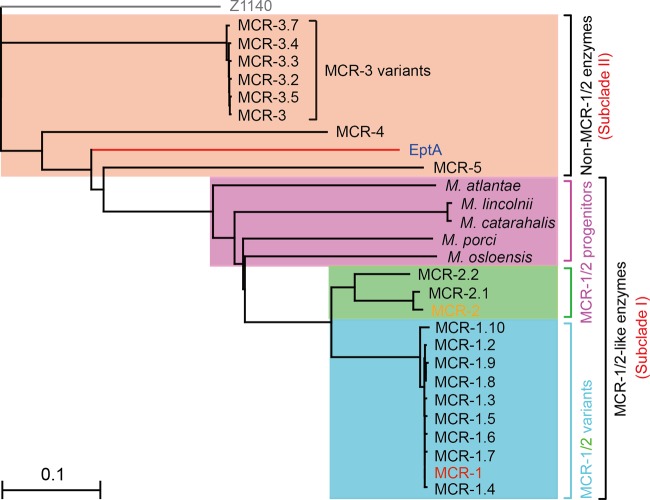
Phylogeny of MCR-like enzymes. All the MCR-like proteins used here were sampled from the protein database of the NCBI website and subjected to phylogenetic analyses using Clustal Omega (https://www.ebi.ac.uk/Tools/msa/clustalo/) (67). The final phylogenetic output was visualized with TreeView (68). The phylogenetic analysis indicates that the MCR-like enzymes are categorized into two groups: subclade I, the MCR-1 and -2-like enzymes, and subclade II, the non-MCR-1 and -2-like enzymes, such as EptA. Subclade I includes 9 MCR-1 variants, 3 MCR-2 variants, and putative MCR-1/-2 progenitors from five different *Moraxella* species (51, 69). The product of the Z1140 locus (in gray) of E. coli O157:H7, a member of the PEA lipid A transferases lacking a role in colistin resistance, is used as an internal reference.

**FIG 4:**
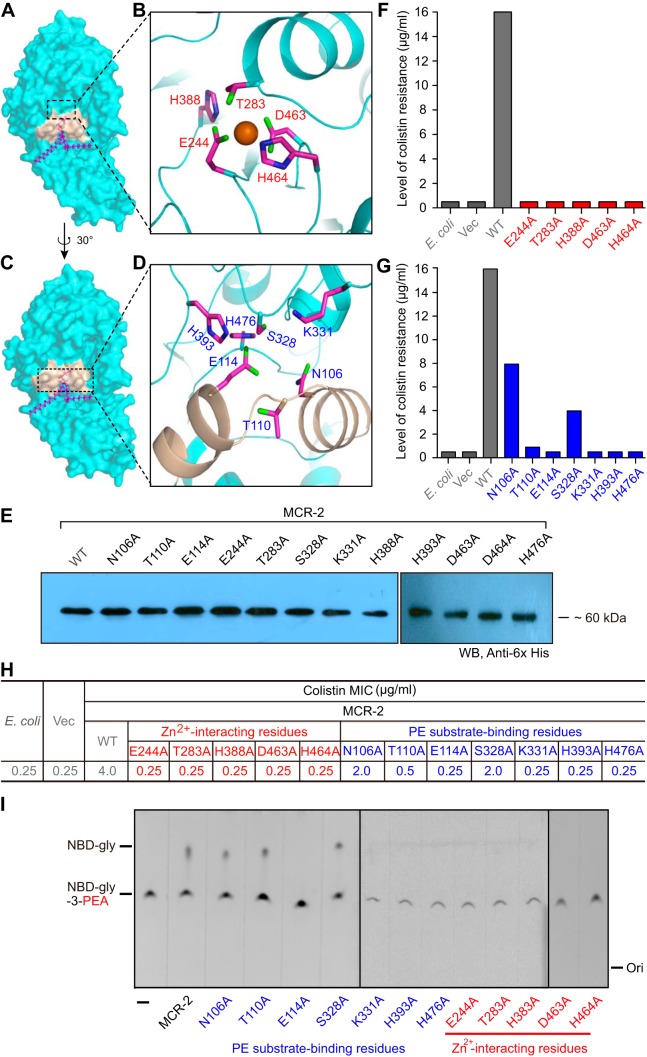
Structure-guided functional analyses of the PE-binding cavity of MCR-2. (A) Surface structure of MCR-2 with the cavity required for entry and binding of PE substrate. (B) Enlarged view of the 5-residue Zn^2+^-binding motif. (C) Surface structure of MCR-2 in the counter clockwise rotation of 30 degrees with fine-structural illustration of the cavity for entry of the PE substrate. (D) Enlarged view of the 7-residue motif with essential roles in the PE-binding cavity of MCR-2. The 7 amino acids (N106, T110, E114, S328, K331, H393, and H476) are proposed to participate in the formation of the PE cavity of MCR-2. (E) Western blotting-based expression analyses of MCR-2 and its 12 point-mutants in E. coli. Given the limit of the wells of PAGE (10 per gel), the photograph of Western blotting here was generated through a combination of two different gel images in which the protein samples were separated. (F) Site-directed mutagenesis assay of the Zn^2+^-binding motif in the context of MCR-2 colistin resistance. The 5 residues in the Zn^2+^-binding motif of MCR-2 are E244, T283, H388, D463, and H464. (G) Site-directed mutagenesis assay of the PE-binding cavity in the context of MCR-2 colistin resistance. The 2 periplasmic-facing helices (in light golden in panel D) possess 3 crucial residues (namely, N106, T110, and E114) that play roles in the binding of MCR-2 to the PE substrate molecule. (H) Comparison of colistin MICs in E. coli strains carrying either wild-type *mcr-2* or its point mutants. (I) TLC-based assays of the enzymatic activities of MCR-2 and its 12 point mutants. Structure-guided site-directed mutagenesis was performed as recommended by the manufacturer. Vec, empty-vector-bearing strain; WT, wild type; Ori, origin. All strains tested here are listed in Table S1.

The phylogeny of the MCR-like proteins indicates that EptA might be evolutionarily distinct from MCR-1 and -2 (Fig. 3), which generally agrees with their differing levels of colistin resistance (Fig. 4; Fig. S2). However, it seems that EptA and MCR-1 have evolved substrate-binding cavities with similar conformations that require identical sets of cavity-forming residues (12 conserved amino acids in total) (Fig. S1 and S2). Based on the assumption that MCR-2 possesses a similar cavity as well, we analyzed its overall structure through superposition of the modeled structure of full-length MCR-2 on that of EptA (Fig. S3). Again, as observed with EptA (45), the overall architecture of MCR-2 contains an N-terminal transmembrane (TM) domain and a periplasm-facing catalytic domain at the C terminus (Fig. S3A). The TM domain, spanning the inner membrane, includes six α-helices (Fig. S3A), and the catalytic domain has a hydrolase fold (Fig. S3B) that consists of 10 α-helices and 7 β-sheets (Fig. S3A). The two domains are connected by four short periplasmic loops (PH2, PH2′, PH3, and PH4), a bridge helix (BH), and a long, coiled loop (Fig. S3A and B). The presence of a long, coiled loop between the BH and β-sheet 1 (S1) facilitates the flexible movement of the catalytic domain to capture PE substrate molecules. Therefore, structural similarities are observed among EptA (45), MCR-1, and MCR-2 (Fig. 2; Fig. S3).

10.1128/mBio.02317-17.3 FIG S3  Structural dissection of MCR-2 defines a conserved cavity required for the PE substrate binding. (A) Topological illustration of the intramembrane protein. (B) Overall structure of the integral membrane protein MCR-2 at full length. (C) Surface structure of the complex of MCR-2 and its PE substrate. (D) Enlarged view of the PE-binding cavity in MCR-2. The catalytic domain is in green, the TM region is in blue, the two helices (PH2 and PH2′) are in light pink, and PE substrate is indicated with sticks. The majority of the cavity region is formed by the PH2 and PH2′ helices plus the TM domain (highlighted with a red arrow). The rectangle with pink background refers to the inner membrane layer. Overall structure of MCR-2 was modeled with the structural template of Neisseria meningitidis EptA (PDB code 5FGN) and depicted in ribbon form using PyMol. TM, transmembrane; PH, periplasmic-facing helices; H, α-helices; S, β-sheet; N, N terminus; C, C terminus; PE, phosphatidylethanolamine. Download FIG S3, JPG file, 0.5 MB. Copyright © 2018 Xu et al. 2018 Xu et al. This content is distributed under the terms of the Creative Commons Attribution 4.0 International license.

Since DDM, a nonionic detergent (Fig. 2A), is only an analogue of the physiological substrate of EptA, PE (Fig. 2B), and is used simply for convenience in performing crystallography, we utilized molecular docking to analyze the true PE substrate by replacing the detergent in EptA-DDM (PDB code 5FGN) (Fig. 2C). This revealed a cavity in EptA for PE substrate entry/binding (Fig. 2D). The energy-scoring component of DOCK 6.7 is based on the implementation of force field scoring. The best score for PE and EptA from docking is −95.11 kcal/mol (the energy of the van der Waals interaction between PE and EptA is −85.25 kcal/mol, and the energy of electrostatic interaction between PE and EptA is −9.86 kcal/mol). Following the structural superposition of EptA and MCR-1, a putative substrate cavity was visualized in MCR-1, accessible by both DDM (Fig. 2E) and PE molecules (Fig. 2F). Based on the modeled MCR-2 structure (Fig. S3C), we observed a similar substrate cavity that can be either occupied by DDM detergent (Fig. 2G) or bound by the PE lipid substrate (Fig. 2H; Fig. S3D). These structural alignments suggest striking similarities in the DDM-/PE-recognizing cavities among EptA, MCR-1, and MCR-2 (Fig. 2C to H), indicating the possibility of parallel biochemical mechanisms for catalytic reactions.

### Functional mapping of the PE-interacting cavity of MCR-2.

Analyses of the MCR-2 surface structure (Fig. 4A and C) allow us to divide the PE-recognizing cavity into two distinct subparts, (i) a 5-residue Zn^2+^-centered motif (E244, T283, H388, D463, and H464) (Fig. 4B) and (ii) a 7-residue PE-interacting domain (N106, T110, E114, S328, K331, H393, and H476) (Fig. 4D). However, functional/experimental evidence validating this is lacking. Thus, we applied site-directed mutagenesis to obtain 12 point mutants of MCR-2 (Fig. 4E) and tested their roles both *in vivo* and *in vitro* (Fig. 4F to I). Prior to functional assays, Western blot analysis demonstrated that all 12 point mutants of MCR-2 are expressed equally as well as the wild-type in E. coli (Fig. 4E). As determined from the results of LBA plate assays, each of the MCR-2 mutants (with 1 of 5 putative Zn^2+^-binding sites mutated) lost the ability to provide colistin resistance (Fig. 4F). Among the 7 point mutants with defects in PE lipid-recognizing sites, 3 MCR-2 mutants retained partial colistin resistance (i.e., those with the N106A [4 μg/ml], S328A [2 μg/ml], and T110A [1 μg/ml] mutations) (Fig. 4G), while the remaining 4 mutants were fully inactive (Fig. 4G). This is generally consistent with colistin MIC measurements (Fig. 4H).

**FIG 5:**
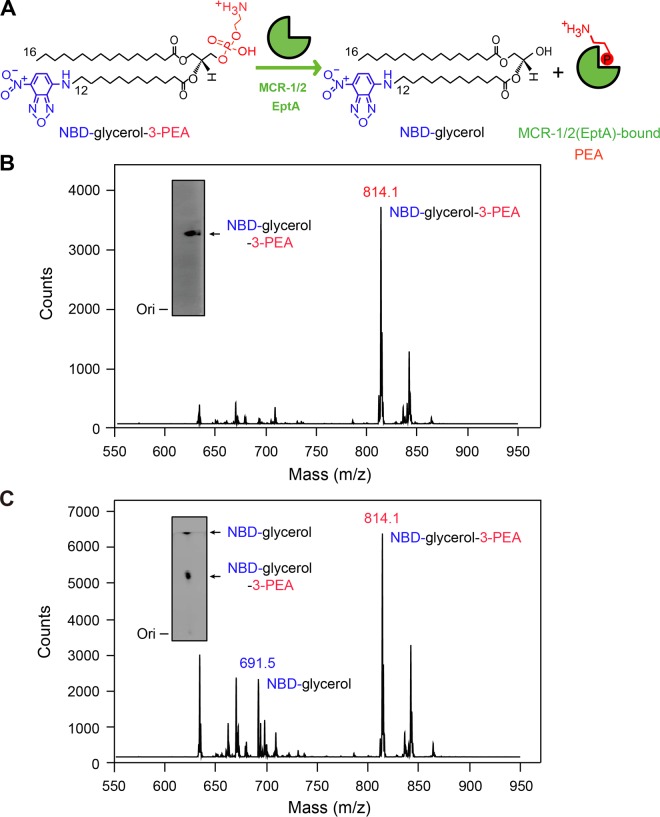
MCR-1 and -2 (and EptA) can release PEA (phosphoethanolamine) from the alternative substrate NBD-glycerol-3-PEA. (A) Scheme for hydrolysis of the alternative substrate NBD-glycerol-PEA into NBD-glycerol and the enzyme-bound PEA adduct. (B) LC-MS identification of NBD-glycerol-3-PEA. The inset gel shows NBD-glycerol-3-PEA separated with thin-layer chromatography (TLC). (C) LC-MS identification of the enzymatic activities of MCR-1/MCR-2/EptA that catalyze cleavage of NBD-glycerol-PEA lipid substrate into NBD-glycerol. The inset gel shows TLC analysis of MCR-1-catalyzed hydrolysis of NBD-glycerol-PEA lipid substrate, giving its product, NBD-glycerol.

**FIG 6:**
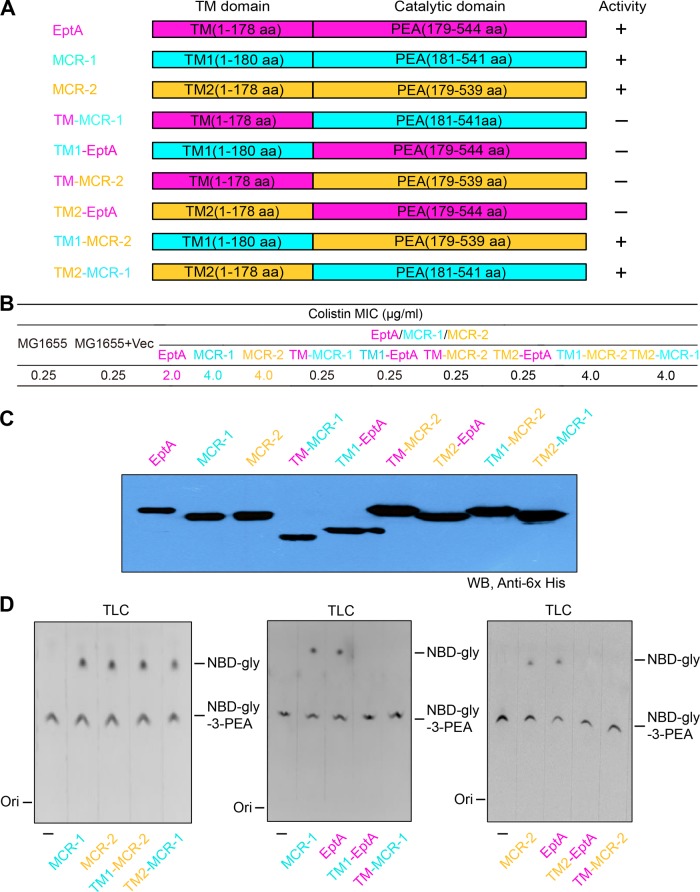
Comparative analyses of hydrolytic activities of MCR-2 and its derivatives. (A) Molecular designs for hybrid versions of MCR-1 and -2 and EptA. +, functional; −, nonfunctional. (B) Colistin MICs of E. coli MG1655 strains expressing *mcr-1* and -*2* and their derivatives. (C) Western blot (WB) analysis of the expression of *mcr-1* and -*2* and their derivatives. Of note, QTOF MS and circular dichroism analyses show that the different migration rates of the chimeric proteins (especially TM-MCR-1 and TM1-EptA) in SDS-PAGE are due not to protein degradation or misfolding but to differing charges. (D) TLC assays of the enzymatic activities of MCR-1 and -2 and their hybrid versions *in vitro*. Domain-swapping analyses suggest that domains are functionally exchangeable between MCR-1 and MCR-2 but not between EptA and MCR-1 or MCR-2. This is consistent with the fact that EptA is evolutionarily distant from MCR-1 and -2, as illustrated in the phylogeny of MCR-like proteins (Fig. 3). TM, transmembrane region of EptA; TM1, transmembrane region of MCR-1; TM2, transmembrane region of MCR-2; PEA, phosphoethanolamine; TLC, thin-layer chromatography; gly, glycerol; NBD-gly-3-PEA, fluorescent label (1-acyl-2-{12-[(7-nitro-2-1,3-benzoxadiazol-4-yl) amino] dodecanoyl}-*n*-glycerol-3-phosphoethanolamine); Ori, origin.

As described for EptA and MCR-1 (Fig. 5 and 6), we also purified all 12 mutant MCR-2 proteins to homogeneity and evaluated the role of the cavity-forming residues in the catalytic activity of MCR-2 using an *in vitro* enzymatic reaction with the fluorescently labeled substrate NDB-glycerol-3-PEA (1-acyl-2-{12-[(7-nitro-2-1,3-benzoxadiazol-4-yl) amino] dodecanoyl}-*sn*-glycero-3-phosphoethanolamine) (Fig. 4I and 5A to C) (45). Thin-layer chromatography (TLC) assays suggested that (i) 3 mutants of MCR-2 (N106A, T110A, and S328A) retain partial enzymatic activities similar to that of the wild-type MCR-2 in that an intermediate product, NBD-glycerol, is produced (Fig. 4I), while (ii) the other 9 MCR-2 point mutants have no detectable activity, as evidenced by NBD-glycerol being absent and only the substrate, NBD-glycerol-3-PEA, being observed, almost identical to the results for the negative controls (Fig. 4I). The differences in the enzymatic activities of MCR-2 mutants were further demonstrated through their ability to modify lipid A anchored on the bacterial outer membrane, as determined using MALDI-TOF MS (Table 1). The presence of wild-type MCR-2 gives a new peak (*m/z*, 1,919.991), corresponding to the production of PPEA-1(4′)-lipid A, when compared to the profile obtained for E. coli MG1655, which has only one dominant lipid A peak (*m/z*, 1,796.243) (Table 1). This validates its role in catalyzing the enzymatic transfer of PE from PE lipids to lipid A *in vivo*. Consistent with observations made during the TLC trials, only 3 of the 12 MCR-2 mutants retained partial PEA transfer activity, giving a specific peak for PPEA-1(4′)-lipid A (*m/z*, 1,919.114 to 1,919.857) (Table 1). The other 9 mutants of MCR-2 lost enzymatic activity because of functional impairment of the cavity occupied by the PE lipid molecule (Table 1). This highlights the importance of cavity-forming residues in the biochemistry of MCR-2, a transferable determinant of colistin resistance, closely matching EptA, an intrinsic determinant of colistin resistance.

### Domain-swapping analyses of EptA, MCR-1, and MCR-2.

Due to the distinct phylogenetic placement of EptA compared to those of MCR-1 and -2 (Fig. 3), we examined the relationship between the two protein domains (transmembrane region and PEA transferase domain) of these enzymes and their relationship with the evolution of protein function in MCR-like enzymes (Fig. 6). Domain swapping was applied in engineering an array of hybrid colistin resistance genes (Fig. 6A). In total, six separate hybrid proteins were constructed, denoted as follows: (i) TM-MCR-1, a modified MCR-1 carrying the TM region of EptA (“TM” in hybrid protein designations); (ii) TM1-EptA, a derivative of EptA with TM replaced by the TM region of MCR-1 (“TM1” in hybrid protein designations); (iii) TM-MCR-2, a modified version of MCR-2 with TM of EptA; (iv) TM2-EptA, a mosaic version of EptA in which TM is replaced with the TM region of MCR-2 (“TM2” in hybrid protein designations); (v) TM1-MCR-2, a derivative of MCR-2 with TM1 of MCR-1; and (vi) TM2-MCR-1, a modified version of MCR-1 where TM2 of MCR-2 is present (Fig. 6A). Subsequently, they were functionally evaluated via colistin MIC measurements. In agreement with our recent observations made with MCR-2 (17), only the two mosaic versions (TM1-MCR-2 and TM2-MCR-1) allow the recipient E. coli cells to exhibit appreciable resistance levels, with a MIC of 4.0 μg/ml (Fig. 6B). Intriguingly, all four derivatives of MCR carrying the TM region of EptA seem to be inactive (colistin MIC of 0.25 μg/ml) and have results indistinguishable from those of the negative controls (Fig. 6B). Western blot analysis demonstrates that all the mosaic proteins are expressed equally as well as the native versions, despite the apparent inconsistency in migration rates in SDS-PAGE (Fig. 6C). Subsequently, we obtained the actual masses of TM-MCR-1 (60.52 kDa) and TM1-EptA (65.85 kDa) via MS and confirmed their sequence identities with ~60% coverage. More importantly, circular dichroism (CD) analyses confirmed that all the hybrid proteins exhibit the typical CD spectra of an α-helix structure (Fig. S4D to G), almost identical to those of EptA (Fig. S4A), MCR-1 (Fig. S4B), and MCR-2 (Fig. S4C). This validates our recent biophysical description of MCR-1 and its derivatives (44). In general consistency with crystallographic observations made by different research groups (38–41, 43), our results from inductively coupled plasma mass spectrometry (ICP-MS) detected the presence of zinc in the full-length MCR-1 and -2 (EptA) proteins, as well as the six mosaic derivatives (Fig. 6A; Fig. S5). These results ruled out the possibility of improper expression/proteolytic degradation of mosaic proteins (Fig. 6C). The inconsistent apparent masses might be due to differences in charge during the separation by SDS-PAGE. This might imply that EptA and MCR-1 and -2 have incompatible TM region and PEA transferase modules (Fig. 6A; Fig. S1), which is at least in part (if not completely) consistent with the scenario that they are evolutionarily distant (Fig. 3).

10.1128/mBio.02317-17.4 FIG S4  Circular dichroism-based secondary structures of EptA, MCR-1 and -2, and their derivatives. (A) CD spectrum of EptA protein. (B) CD profile of MCR-1. (C) CD analyses of MCR-2. (D) Use of CD to visualize secondary structure of EptA derivative, TM1-EptA. (E) CD spectrum of MCR-1 derivative TM-MCR-1. (F) CD-based elucidation of EptA derivative TM2-EptA. (G) CD-aided identification of MCR-2 derivative TM-MCR-2. CD, circular dichroism; TM1-EptA, a derivative of EptA whose TM region is replaced with its counterpart in MCR-1 (panel B); TM-MCR-1, a derivative of MCR-1 whose TM region is replaced with its counterpart in EptA (panel A); TM2-EptA, a derivative of EptA whose TM region is replaced with its counterpart in MCR-2 (panel C); TM-MCR-2, a derivative of MCR-2 whose TM region is replaced with its counterpart in EptA (panel A). Download FIG S4, JPG file, 0.9 MB. Copyright © 2018 Xu et al. 2018 Xu et al. This content is distributed under the terms of the Creative Commons Attribution 4.0 International license.

10.1128/mBio.02317-17.5 FIG S5  ICP-MS analyses of Zn^2+^ in EptA, MCR-1 and -2, and the four chimeric derivatives (TM-MCR-1, TM1-EptA, TM2-EptA, and TM-MCR-2). Binding of zinc to diversified MCR-like proteins was measured using inductively coupled plasma mass spectrometry (ICP-MS) as described by Loeschner et al. (57). ZnCl_2_ was used as the positive control. Download FIG S5, TIF file, 2 MB. Copyright © 2018 Xu et al. 2018 Xu et al. This content is distributed under the terms of the Creative Commons Attribution 4.0 International license.

**FIG 7:**
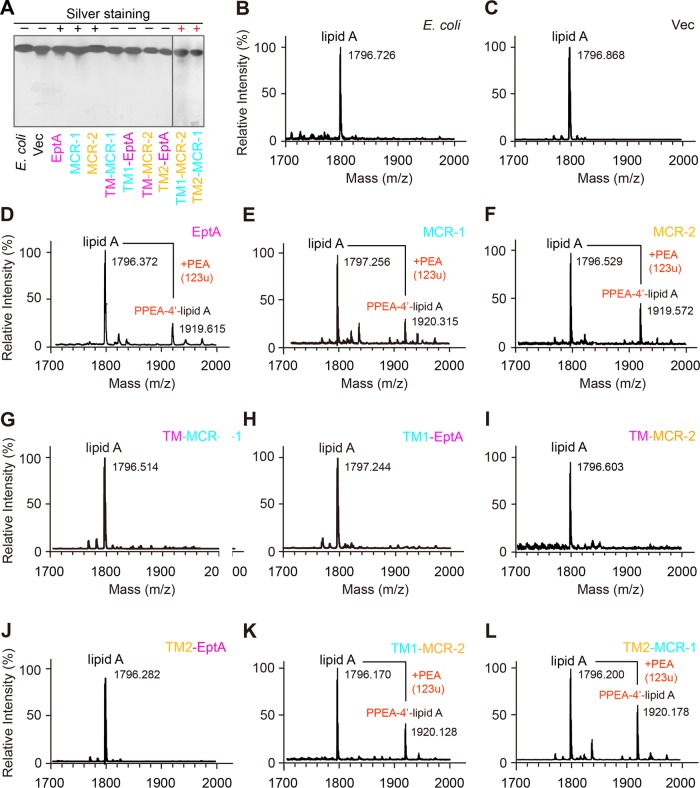
MS determination of altered structures of LPS-lipid A anchored on the bacterial surface upon the expression of MCR-1 and -2 and its derivatives. (A) Silver-staining analysis of LPS-lipid A isolated from diverse E. coli strains with or without *mcr-1* or -*2* or their derivatives. +, lipid A with the addition of PEA; −, intact lipid A; Vec, empty vector. (B, C) LPS-lipid A profiles of the two negative-control strains, E. coli MG1655 alone (B) and MG1655 with the empty vector pBAD24 (C). (D to F) Appearance of a unique peak for the modified lipid A (PPEA-4′-lipid A) in the E. coli strains expressing any one of the three genes *eptA* (D), *mcr-1* (E), and *mcr-2* (F). (G, H) No modification of lipid A by the expression of the MCR-1 hybrid (TM-MCR-1) whose transmembrane region is replaced by its counterpart from EptA (G) or the reverse hybrid, TM1-EptA (H). (I, J) MALDI-TOF MS suggests only an unmodified lipid A peak is present in the MG1655 strain regardless of the presence of TM-MCR-2 (I) or TM2-EptA (J). (K, L) Identification of a modified lipid A, PPEA-4′-lipid A, in the E. coli strains harboring either TM1-MCR-2 (K) or TM2-MCR-1 (L). LPS, lipopolysaccharide; TM-MCR-2, a derivative of MCR-2 in which the transmembrane region is replaced by the counterpart from EptA; TM2-EptA, a hybrid version of EptA carrying the transmembrane region from MCR-2; TM1-MCR-2, a derivative of MCR-2 with the MCR-1 transmembrane region; TM2-MCR-1, a derivative of MCR-1 with the MCR-2 transmembrane region. The MS peak of lipid A species in E. coli occurs at *m/z* of 1,796.170 to 1,797.256, whereas it appears at *m/z* 1,919.615 to 1,920.315 in E. coli cells with functional expression of MCR-1 and -2, EptA, and its derivatives, because the modified PEA species [PPEA-1(4′)-lipid A] is present. Here, the PEA mass is 123 unified atomic mass units (u).

As described by Anandan et al. (45), we further assayed the enzymatic activities of EptA/MCR and derivatives *in vitro* (Fig. 4I, 5, and 6D). It is thought that EptA-/MCR-originated enzymes can catalyze the cleavage of the PEA moiety from the alternative substrate NBD-glycerol-3-PEA (Fig. 5A and B), giving a fluorescently tagged product, NBD-glycerol (Fig. 5A to C). As expected, TLC experiments showed the following: (i) the NBD-glycerol product band consistently appears upon the addition of EptA/MCR-1/MCR-2 protein in DDM micelles, whose migration rate is faster than that of the substrate NBD-glycerol-3-PEA (Fig. 6D); (ii) the two hybrid versions of MCR-1 and MCR-2 (TM1-MCR-2 and TM2-MCR-1) retain hydrolytic activity on NBD-glycerol-3-PEA (Fig. 6D); and (iii) the four mosaic forms of MCR with EptA modules inserted lose the enzymatic ability to transfer the PEA moiety (Fig. 6D). Of note, both the NBD-glycerol-3-PEA substrate (Fig. 5B) and its resultant NBD-glycerol product (Fig. 5C) were verified by liquid chromatography-mass spectrometry (LC-MS). Driven by the fact that an intermediate of PEA-threonine 280 (T280)-EptA is present in the soluble EptA of Neisseria meningitidis (24), we speculated that PEA might be released as an adduct of PEA-T285-MCR-1 (equivalent to PEA-T283-MCR-2) (Fig. 1B and 5A). Subsequently, MALDI-TOF MS analyses of lipid A species were performed to trace the *in vivo* transfer of the PEA moiety from its physiological donor phosphatidylethanolamine (PE) to the acceptor LPS-lipid A (Fig. 1C and 7). In addition to the single lipid A peak seen in negative controls (Fig. 7A to C), another peak [PPEA-1(4′)-lipid A] consistently appears in the E. coli strains expressing *eptA*, *mcr-1*, and *mcr-2* (Fig. 7D to F). Of the strains expressing the six mosaic proteins, only two (expressing TM1-MCR-2 and TM2-MCR-1) show a new peak (*m/z* of 1,920.128 for TM1-MCR-2 [Fig. 7K] and 1,920.178 for TM2-MCR-1 [Fig. 7L]) with a mass shift corresponding to the addition of a PEA group, whereas the strains expressing the other four hybrid versions (TM-MCR-1, TM1-EptA, TM-MCR-2, and TM2-EptA) do not (Fig. 7G to J). These MS results match the scenarios seen with TLC enzymatic trials *in vitro* (Fig. 6D) and assays for colistin resistance (Fig. 6B). In summary, these data suggest that protein evolution has rendered a functional differentiation among EptA, MCR-1, and MCR-2 (Fig. 3 to 7).

## DISCUSSION

Since MCR-1-mediated colistin resistance is spreading globally and is becoming a serious risk for human health, colistin will be formally banned as an animal growth booster in China. It is now being reconsidered for human clinical applications as well. In general, colistin resistance is physically dependent on bacterial surface remodeling, which interferes with (and even impairs) static-electron interaction between the cationic antibiotic peptide and its primary target, the negatively charged lipid A moiety of LPS species. Chemical modifications of the lipid A moiety anchored to the outer leaflet of the outer membrane of Gram-negative bacteria decrease the net negative charge of the surface and confer colistin resistance (Fig. 1).

Although MCR-2 was earlier suggested to confer an appreciably stronger resistance to colistin than MCR-1 in E. coli, our data here show that three colistin resistance proteins (EptA, MCR-1, and MCR-2) share a similar PE lipid substrate-recognizing cavity (Fig. 2 and 4; Fig. S2). This parallel nature of the cavities of these three protein allows them to exploit an identical mechanism for enzymatic catalysis (Fig. 1). In fact, in addition to EptA, which according to Anandan et al. (45) utilizes a possible ping-pong mechanism of catalysis, our biochemical data confirm that MCR-2 (and/or MCR-1) might also do so. This is, probably, a common machinery among the family of PEA lipid A transferases, since they consistently share the same cavity, consisting of 12 essential residues, for the entry of the PE lipid substrate molecule. In addition to the structural insights gained from the cavity of MCR-1 (Fig. 2), we utilized a structure-guided functional mapping approach to finely dissect the physiological roles of 12 core residues of the PE binding cavity in both EptA and MCR-2 (Fig. 4; Fig. S2 and S3). The domains (TM region and PEA transferase) of the closely related MCR-1 and MCR-2 are functionally exchangeable (Fig. 6 and 7), while those of EptA and MCR-1 (and/or MCR-2) are not (Fig. 6 and 7), implying structural variations among PE-occupied cavities. Considering the lower level of similarity between EptA and MCR-1 (and/or MCR-2) (Fig. S1), we believe that EptA might represent an ancestral variant of MCR-1 (MCR-2). Indeed, this hypothesis is also supported by the evolutionary enhancement of the colistin resistance of MCR-1/MCR-2 (MIC of 4 μg/ml) (Fig. 4 and 6B) compared to that of EptA (MIC of 2 μg/ml) (Fig. 6B; Fig. S2).

In addition to the chromosomally encoded colistin resistance protein EptA, two plasmid-borne PEA transferases (MCR-1 [46] and MCR-2 [16, 17], with 81% amino acid identity) that can confer colistin resistance to recipient bacterial strains have already been identified. Thus, it seems very plausible that the rapid and global dissemination of colistin resistance might proceed not in the way of clonal expansion (intrinsic resistance), but through plasmid-based conjugative transfer (transferable resistance) (27). In particular, several genetic variants of *mcr-1* with point mutations have been detected, including *mcr-1.2* (47), *mcr-1.3* (48), *mcr-1.5* (49), and *mcr-1.6* (50). This highlights the possibility that *mcr-1* is under antibiotic selection pressure. Since MCR-3 (558 amino acids) (31), another recently discovered PEA transferase, exhibits closer similarity to EptA (53.1% amino acid similarity and 36.4% identity) than it does to MCR1 and MCR 2 (44.1% and 45.2% similarity and 32.2% and 31.3% identity, respectively), we tend to believe that MCR-3 and other MCR proteins, like MCR-4 and MCR-5, might be EptA-like enzymes rather than MCR-1 and -2-like proteins (Fig. 3). This speculation is validated by the comparable levels of colistin resistance obtained in our trials (8 μg/ml on LBA plates for both EptA [Fig. S2F and G] and MCR-3 [not shown]). Moreover, Kieffer and coworkers also reported an MCR-like gene from Moraxella osloensis (AXE82_07515) encoding a 548-amino-acid product with 73.6% and 75.2% similarity (61.4% and 63.1% identity) to MCR-1 and MCR-2, respectively (51). The product of this MCR-like gene, AXE82_07515, only possesses 44.3% similarity and 29.1% identity to MCR-3, which might indicate a growing confusion in the nomenclature used for MCR-like genes. It is probable that *mcr-3* and AXE82_07515 both might be ancestors/intermediates for *mcr-1* and *-2* genes, though this hypothesis needs further experimental evidence. Also, the convergent/divergent nature of the evolution of this branch of MCR-like enzymes (MCR-3, -4, and -5) from MCR-1 and -2 is unexplored. Unexpectedly, another member of the PEA transferase family, encoded by locus Z1140 (539-amino-acid product) in E. coli O157:H7 strain EDL 933 (accession number AE005271), did not play any detectable role in our trials of enzymatic activity and colistin resistance (not shown), although it displayed 54.3%, 46.8%, 47.3%, and 64.3% similarity (33.6%, 34.7%, 52.5%, and 36.2% identity) to EptA, MCR-1, MCR-2, and MCR-3, respectively (Fig. 3; Fig. S1). The protein encoded by Z1140 might be a relic of the developing or degenerative family of PEA lipid A transferases.

## MATERIALS AND METHODS

### Strains, plasmids, and growth conditions.

All E. coli strains and plasmids used here are listed in Table S1 in the supplemental material (52). The two strains DH5α and BL21(DE3)pLysS were used for DNA manipulation and protein expression, respectively. E. coli MG1655 was used a recipient for *eptA*, *mcr-1* and -*2*, and their mutants. To create hybrid genes of *eptA* and *mcr-1* and -*2*, overlapping PCR was performed as described by Sun et al. (17) with appropriate sets of specific primers (Table S2). The Mut Express II fast mutagenesis kit version 2 (Vazyme Biotech Co., Ltd.) with proper primers (Table S2) was used to generate an array of *eptA* and/or *mcr-2* mutants, in which pBAD24::*eptA* and/or pBAD24::*mcr-2* acted as a template. The isopropyl-β-d-thiogalactopyranoside (IPTG)-inducible pET21a expression vector and arabinose-inducible plasmid pBAD24 (Table S1) were utilized for protein expression and functional assays, respectively, for EptA and/or MCR-1 and -2 or its derivatives. All the recombinant plasmids were verified by direct DNA sequencing. Both liquid Luria-Bertani (LB) broth and solid LB agar (LBA) plates were used to maintain E. coli cultures, and appropriate antibiotics, such as kanamycin and colistin, were supplemented when necessary.

10.1128/mBio.02317-17.6 TABLE S1  Strains and plasmids in this study. Download TABLE S1, DOC file, 0.1 MB. Copyright © 2018 Xu et al. 2018 Xu et al. This content is distributed under the terms of the Creative Commons Attribution 4.0 International license.

10.1128/mBio.02317-17.7 TABLE S2 Primers used in this study. Download TABLE S2, DOC file, 0.1 MB. Copyright © 2018 Xu et al. 2018 Xu et al. This content is distributed under the terms of the Creative Commons Attribution 4.0 International license.

### Measurement of colistin resistance.

The MIC of colistin was determined using a liquid broth dilution test with cation-adjusted Mueller-Hinton broth (CAMHB) as recommended by EUCAST (17). First, an overnight culture from a single colony was diluted 100-fold in fresh CAMHB medium and collected for determination of the MIC when its optical density at 600 nm (OD_600_) reached 0.5. Subsequently, the cultures were diluted to an OD_600_ of 0.05 in CAMHB medium supplemented with various levels of colistin (0, 0.25, 0.5, 1.0, 2.0, 4.0, 8.0, and 16.0 μg/ml) and incubated with shaking at 200 rpm at 37°C for 16 h. The colistin MIC was recorded using optical density measurements. A concentration of 0.2% arabinose was added into CAMHB medium to induce the expression of pBAD24-borne *eptA* and/or *mcr-1* and -*2* and their derivatives in E. coli.

In addition, the survival ability of E. coli expressing *eptA* and/or *mcr-1* and -*2* and their derivatives was also assayed using a solid LBA dilution test (25, 43). Briefly, mid-log-phase cultures diluted appropriately were spotted on LBA plates with various levels of colistin (0, 0.5, 1.0, 2.0, 4.0, 8.0, 16.0, and 32.0 μg/ml). To judge the formation of colonies, these LBA plates were incubated at 37°C overnight and supplemented with 0.2% arabinose to promote protein expression.

### Expression and purification of membrane proteins.

One-liter amounts of mid-log-phase cultures of bacteria encoding MCR-1 and -2, EptA, and their variants (OD_600_ of 0.8 to 1.0) were induced with 0.5 mM IPTG overnight in a shaker (220 rpm) at 18°C. Bacterial cells were centrifuged (5,000 rpm for 25 min) at 4°C prior to washing with 1× phosphate-buffered saline (PBS) (17). The cell pellets were resuspended in buffer A (20 mM Tris-HCl [pH 8.0], 100 mM NaCl, 5 mM DNase I, 1 mM phenylmethylsulfonyl fluoride [PMSF], 2 mM MgCl_2_) to 20% (wt/vol) and then lysed by passage through a French press (JN-Mini, China) at 500 lb/in^2^ once and 1,300 lb/in^2^ twice. Following a round of centrifugation (16,800 rpm for 1 h at 4°C), the resultant supernatant was subjected to further centrifugation (38,000 rpm for 1 h at 4°C). The pellet contained the membrane protein of interest (17, 43).

The pellet was solubilized in buffer B (20 mM Tris-HCl [pH 8.0], 100 mM NaCl, 5% glycerol, 1% DDM [wt/vol]) and centrifuged at 38,000 rpm for 1.5 h at 4°C, and the resultant supernatant was collected for incubation with preequilibrated Ni-nitrilotriacetic acid (NTA) agarose beads overnight at 4°C. Subsequently, the Ni-NTA agarose beads were loaded on a column and rinsed with wash buffer (20 mM Tris-HCl [pH 8.0], 100 mM NaCl, 30 mM imidazole, 5% glycerol, 0.03% DDM [wt/vol]), and the target membrane proteins (EptA, MCR-1, MCR-2, and their mutant versions) were eluted using an elution buffer (20 mM Tris-HCl [pH 8.0], 100 mM NaCl, 100 mM imidazole, 5% glycerol, 0.03% DDM [wt/vol]) (17, 43). In total, 39 membrane proteins were prepared, including wild-type EptA, MCR-2, and MCR-1 proteins and 12 point mutants of each.

### LC-QTOF MS.

The identities of the four chimeric proteins of MCR-1 and -2 (TM-MCR-1, TM1-EptA, TM-MCR-2, and TM2-EptA) were examined using a Waters quadrupole time of flight (QTOF) API-US mass spectrometer (53, 54). A band obtained from SDS-PAGE separation of purified protein was digested with trypsin (G-Biosciences St. Louis, MO), and the resultant peptides were analyzed with a Waters Atlantis C_18_ column (0.03-mm particle size, 0.075 by 150 mm). Finally, the acquired data were subjected to further analyses through the Waters ProteinLynx Global Server 2.2.5, Mascot (Matrix Sciences), and BLAST against the NCBI nr database.

### CD analyses.

In addition to the three parental enzymes EptA, MCR-1, and MCR-2, four hybrid proteins, namely, TM1-EptA, TM-MCR-1, TM2-EptA, and TM-MCR-2, were analyzed in circular dichroism (CD)-based protein-folding trials. For each CD trial, 600 μl of protein (approximately 0.2 mg/ml) in Tris buffer (20 mM Tris-HCl, 300 mM NaCl, 0.03% DDM, 10% [vol/vol] glycerol [pH 8.0]) was measured as we very recently described (44). The CD spectra were collected on a Jasco model J-1500 spectrometer (Jasco Corp., Tokyo, Japan) by continuous wavelength scanning (in triplicate) from 200 to 260 nm at a scan rate of 50 nm/min (55) and smoothed with a Savitsky-Golay filter (56).

### ICP-MS.

As recently performed by us (44), with minor changes, inductively coupled plasma mass spectrometry (ICP-MS) was adopted to detect the presence of zinc ions in MCR-1, MCR-2, EptA, and their derivatives. The protein samples (~0.2 mg/ml) were analyzed using a NexION 300 ICP-MS instrument (PerkinElmer, USA) switched to collision-cell mode. The mass-to-charge ratio (*m/z*) was quantified using the kinetic energy discrimination (KED) mode with helium as the carrier gas (57).

### *In vitro* enzymatic reaction for EptA and MCR-1 and -2.

To visualize the ability of MCR-1, MCR-2, EptA, and their derivatives to remove PEA from a PE lipid substrate, an *in vitro* enzymatic reaction was established as described by Anandan et al. (45), using the fluorescent substrate NBD-PEA (1-acyl-2-{12-[(7-nitro-2-1,3-benzoxadiazol-4-yl) amino] dodecanoyl}-*sn*-glycero-3-phosphoethanolamine; Avanti Lipids, United States). The reaction mixture (comprising 50 mM HEPES [pH 7.50], 100 mM NaCl, 0.03% DDM, 0.2 mM NBD-PEA, and 40 μM EptA, MCR-1, or MCR-2 in a total volume of 50 μl) was incubated overnight at 25°C (44). Thin-layer chromatography (TLC) was used to distinguish the product, NBD-glycerol, from the remaining substrate, NBD-glycerol-3-PEA, in the aforementioned enzymatic reaction mixture. After prerunning the silica TLC plates once in the running buffer (ethyl acetate-methanol-water, 7:2:1 [vol/vol]), TLC-based separation was conducted and the fluorescence signal was detected by Epi blue light (455 to 485 nm) and a corresponding filter in the ChemiDoc MP imaging system (Bio-Rad, CA) (45).

### LC-MS.

As in our recently conducted experiments (44), the identities of both the substrate of NBD-glycerol-3-PEA and the product of NBD-glycerol (from the MCR-1-catalyzed half reaction) were determined by using a liquid chromatography-mass spectrometry (LC-MS) system (Agilent Technologies 6460 triple-quadrupole LC-MS) (58). The electrospray ionization (ESI) source was connected with the mass spectrometer, and the neutral loss ion (*m/z*, 141) mode was set to scan the positive ion. Using a Zorbax SB C_18_ analytic chromatography column (2.1 by 50 mm, 3.5 μm), the samples were eluted with a solution of methanol–0.1% methanoic acid (95:5) at 0.3 ml/min.

### Extraction, purification, and identification of LPS-lipid A.

LPS-lipid A was extracted routinely according to previously established protocols (24, 59, 60). First, bacterial cultures grown on LBA plates with an appropriate level of colistin were stripped and washed with washing buffer containing 30 mM Tris-HCl. Lysozyme was added to the bacterial cells (suspended in a buffer of 30 mM Tris-HCl and 20% sucrose) to disrupt the cell wall. The lysates were dissolved in 3 mM EDTA and subjected to further sonication. Next, the crude LPS was precipitated following the removal of cell debris by 1 h of centrifugation at 16,000 rpm. The crude LPS was resuspended in 30 mM Tris-HCl and 0.2% SDS and subjected to successive treatments of DNase I (25 µg/ml), RNase A (100 µg/ml) at 37°C for 1 h and proteinase K at 37°C for 1 h. The reaction was stopped by incubation at 100°C for 1 h. The purified LPS-lipid A was acquired after the removal of SDS contaminants by two rounds of washing with acidified ethanol and 95% ethanol. The purified lipid A species in LUG loading buffer [250 mM Tris-HCl (pH 6.8), 10% (vol/vol) SDS, 1% bromophenol blue, 10% (vol/vol) glycerol, 5% (vol/vol) 2-mercaptoethanol] were visualized with SDS-PAGE (10%) coupled with silver staining, and analyzed by MALDI-TOF MS (ultrafleXtreme; Bruker) in negative ion mode with the linear detector (24, 59, 60).

### Bioinformatics, structural modeling, and molecular docking.

Sequence alignment of PEA lipid A transferases was conducted with Clustal Omega (https://www.ebi.ac.uk/Tools/msa/clustalo/) and processed via the program ESPript 3.0 (http://espript.ibcp.fr/ESPript/cgi-bin/ESPript.cgi) (61). The transmembrane regions of EptA and MCR-1 and -2 were predicted using TMHMM server version 2.0 (http://www.cbs.dtu.dk/services/TMHMM/). The structure of MCR-2 was modeled using Swiss-Model software (62), in which *N. meningitis* EptA (PDB code 5FGN) acted as the structural template (45). MCR-2 (MCR-1) exhibits 35.4% (35.6%) identity to EptA, and its modeled structure possesses a coverage score of 96% (amino acids 10 to 538) compared with that of EptA. The global model quality estimation (GMQE) score is 0.7, and the QMEAN value (which provides a global and local absolute quality estimate for the modeled structure [63]) is −4.02, implying a reliable qualified structural prediction. The ready-to-dock chemical structure of PE (ZINC identification number [ID] ZINC32837871) and head group of PE (ZINC ID ZINC02798545) were sampled from the ZINC database (64). UCSF DOCK 6 software (version 6.7) was applied to predict binding patterns of the PE molecule versus EptA and the head group of PE to MCR-1 and MCR-2 (65). LigPlot+ was used to illustrate the diagrams for possible ligand-protein interaction (66).

### Phylogenetic analyses.

A collection of MCR-like proteins were aligned using Clustal Omega (https://www.ebi.ac.uk/Tools/msa/clustalo/) (67). The phylogenetic tree was generated and visualized using TreeView (68). In addition to EptA, the MCR-like proteins included the following: MCR-1 and its 8 variants (46); MCR-2 (16) plus its 2 variants, MCR-2.1 (accession number ASK49941) and MCR-2.2 (accession number ASK49942) (69); 5 putative MCR-1 and -2 progenitors, including AXE82_07515 of Moraxella osloensis (accession number WP_082741435) and the counterparts in Moraxella atlantae (accession number WP_082987868), Moraxella lincolnii (accession number WP_078308297), Moraxella catarrhalis (accession number WP_081259431), and Moraxella porci (accession number WP_078317642) (51); MCR-3 (accession number NG_055505) (31), MCR-3.2 (accession number NZ_FLWO01000034) (31), MCR-3.3 (accession number NZ_FLXA01000011) (31), MCR-3.4 (accession number NG_055497), MCR-3.5 (accession number ERR1971735) (32), and MCR-3.7 (accession number MF489760) (33); MCR-4 (accession number MF543359) (36); and MCR-5 (accession number KY807921) (37). Of note, the heterogeneous variants of MCR-1 are separately denoted as MCR-1.2 (accession number WP_065274078) (47), MCR-1.3 (accession number WP_077064885) (48), MCR-1.4 (accession number WP_076611062), MCR-1.5 (accession number ARX60875) (49), MCR-1.6 (accession number WP_077248208) (50), MCR-1.7 (accession number WP_085562392), MCR-1.8 (accession number WP_085562407), and MCR-1.9 (accession number KY964067) (46). The protein encoded by locus Z1140 (accession number AAG55285), a putative PEA transferase of E. coli O157:H7 strain EDL 933, was used as an internal reference in the phylogenetic tree.
