# Supplementary figures and images for: An Evolutionarily Conserved Mechanism for Intrinsic and Transferable Polymyxin Resistance
Source: mBio. 2018 Apr 10;9(2):e02317-17. doi: 10.1128/mBio.02317-17 (PMC5893884; doi:10.1128/mBio.02317-17)

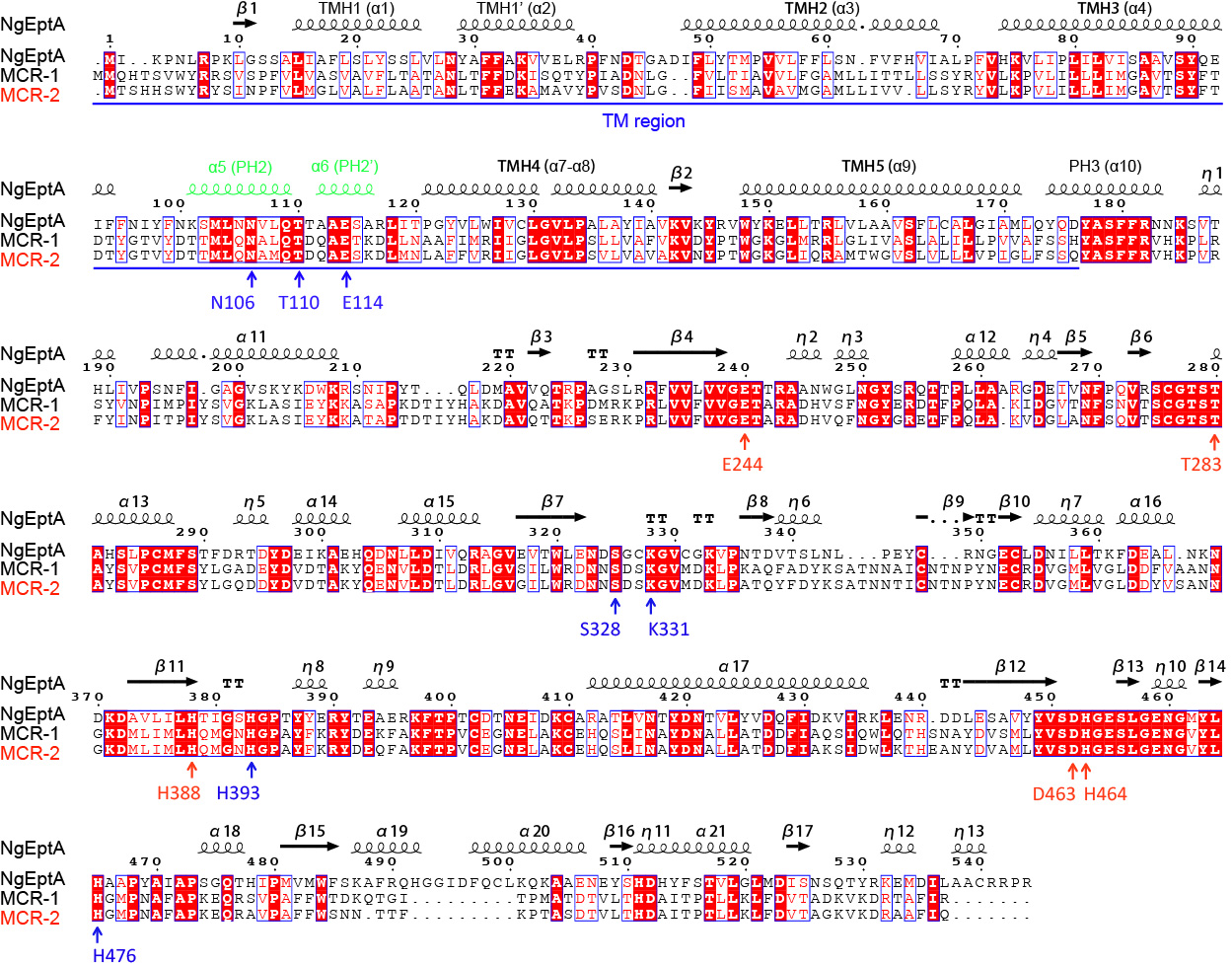

Supplement: FIG S1 [file mbo002183817sf1.jpg]

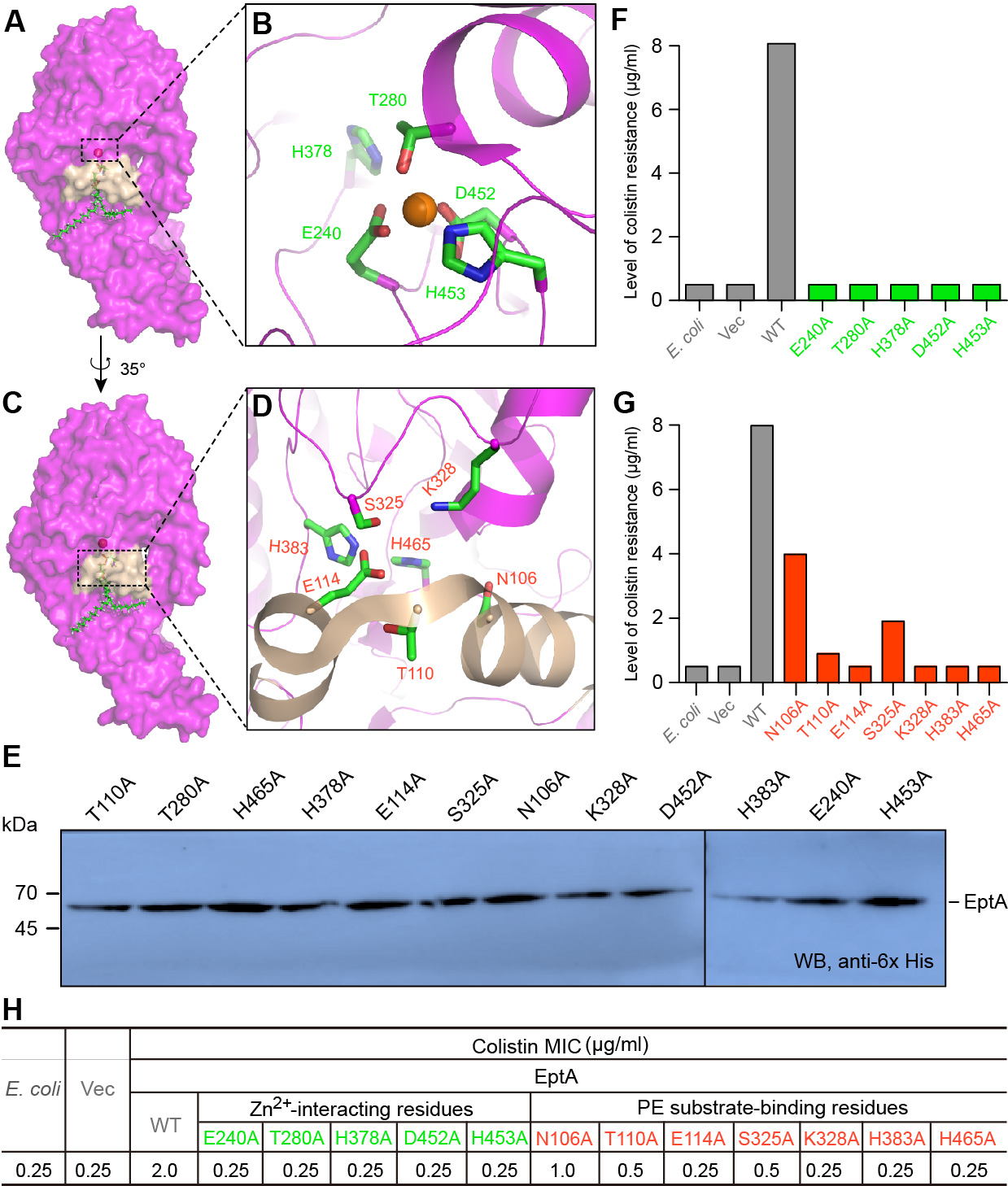

Supplement: FIG S2 [file mbo002183817sf2.jpg]

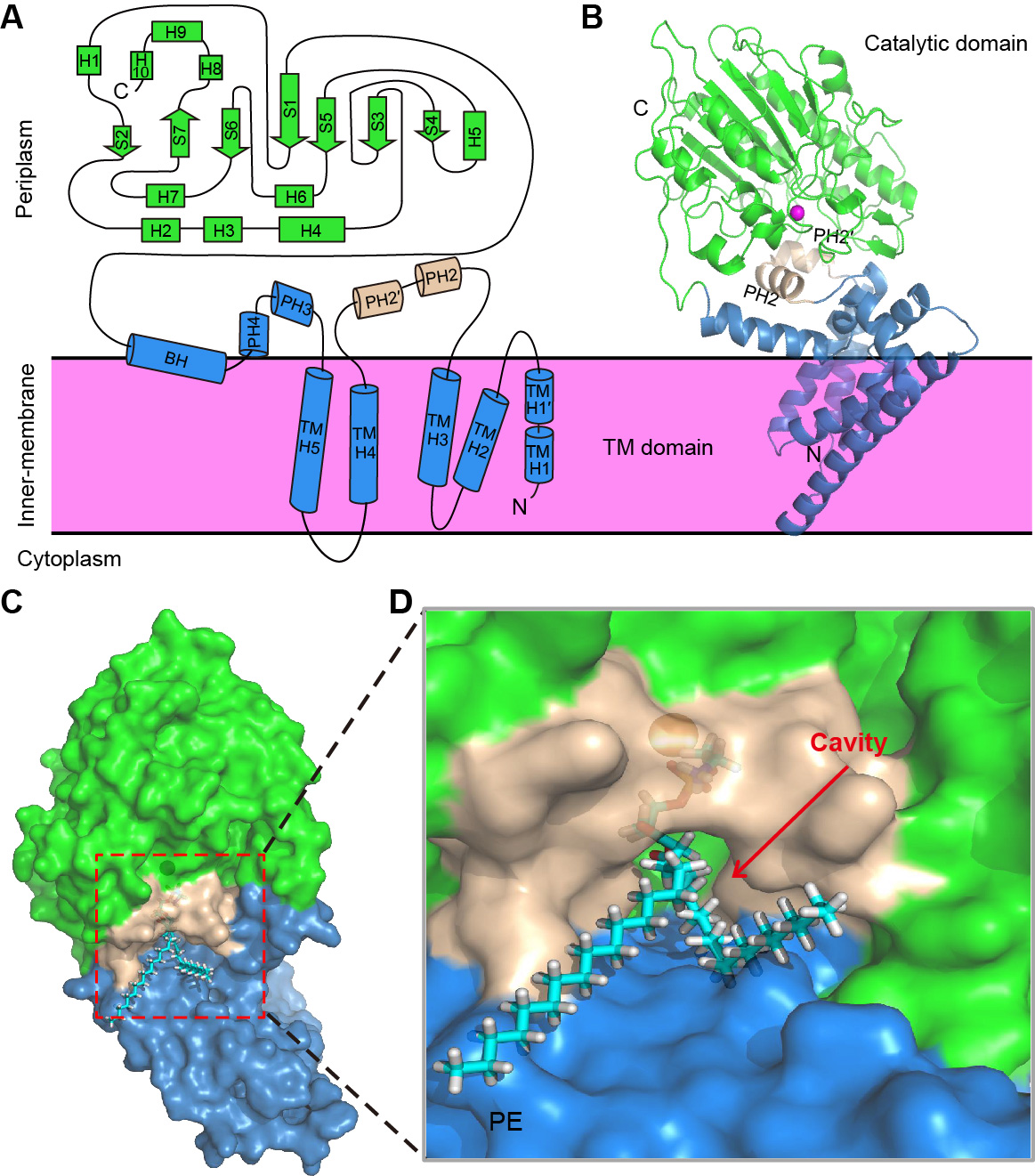

Supplement: FIG S3 [file mbo002183817sf3.jpg]

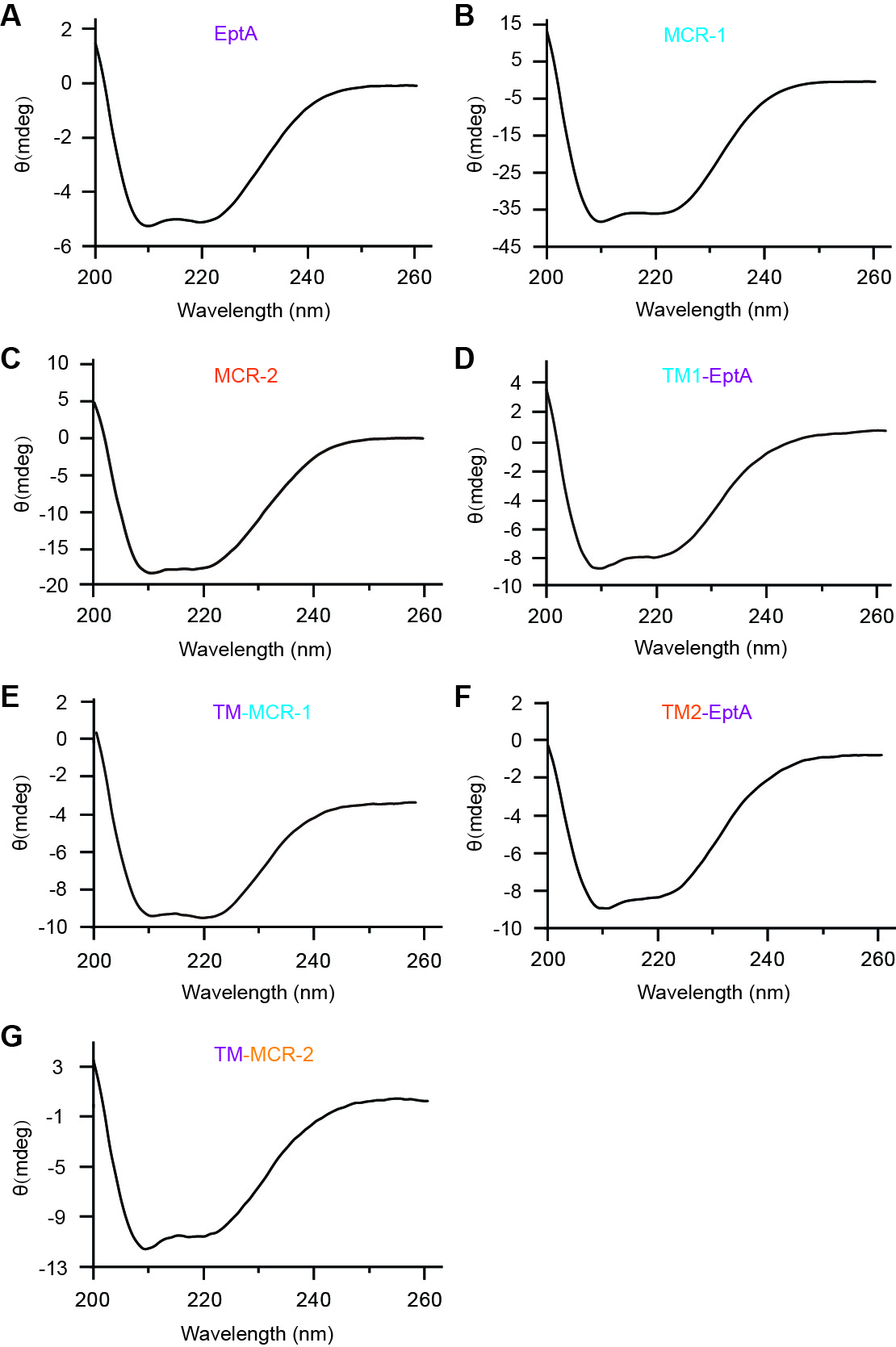

Supplement: FIG S4 [file mbo002183817sf4.jpg]

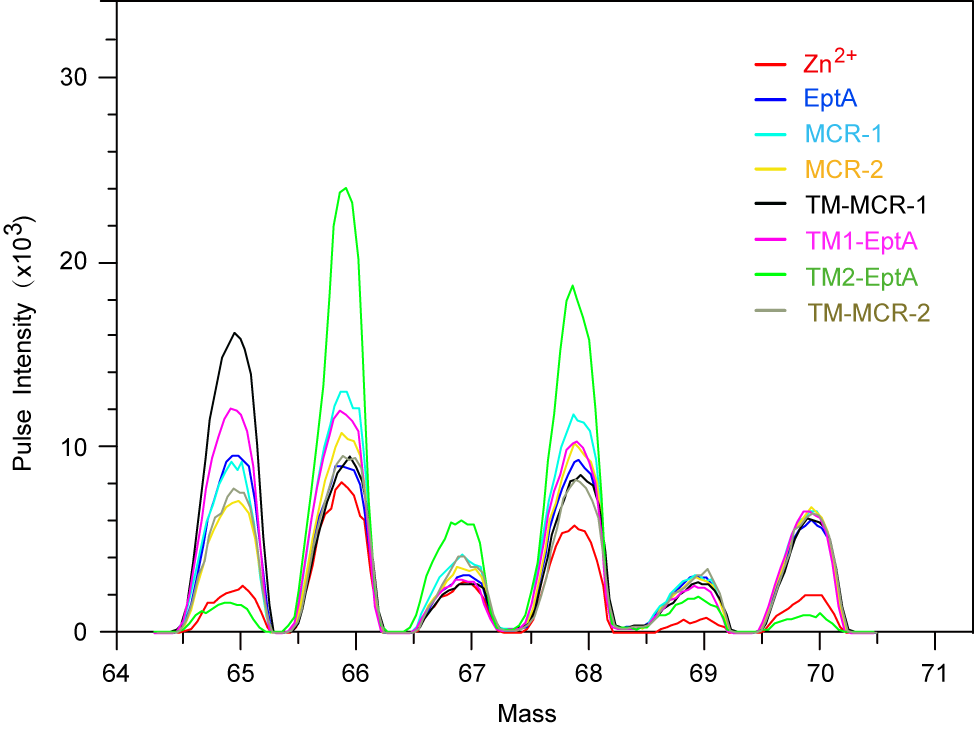

Supplement: FIG S5 [file mbo002183817sf5.tif]
