# Supplementary material for: An Evolutionarily Conserved Mechanism for Intrinsic and Transferable Polymyxin Resistance
Source: mBio. 2018 Apr 10;9(2):e02317-17. doi: 10.1128/mBio.02317-17 (PMC5893884; doi:10.1128/mBio.02317-17)
Supplement: TABLE S1 [file mbo002183817st1.doc]

**Table S1** Bacteria and plasmids used in this study

| Strain or plasmids | Relevant characteristics | Origins |
| --- | --- | --- |
| Strains | | |
| DH5α | A cloning host of *E. coli* | Lab stock |
| MG1655 | A wild-type strain of *E. coli* | Lab stock |
| FYJ795 | MG1655 carrying pBAD24*::mcr-1* | Lab stock |
| FYJ796 | MG1655 carrying pBAD24 | Lab stock |
| FYJ832 | MG1655 carrying pBAD24*::eptA* | Lab stock |
| FYJ855 | MG1655 carrying pBAD24*::mcr-2* | Lab stock |
| FYJ906 | MG1655 carrying pBAD24*::mcr-2*(E244A) | Lab stock |
| FYJ907 | MG1655 carrying pBAD24*::mcr-2*(T283A) | Lab stock |
| FYJ908 | MG1655 carrying pBAD24*::mcr-2*(H393A) | Lab stock |
| FYJ909 | MG1655 carrying pBAD24*::mcr-2*(D463A) | Lab stock |
| FYJ910 | MG1655 carrying pBAD24*::mcr-2*(H464A) | Lab stock |
| FYJ911 | MG1655 carrying pBAD24*::mcr-2*(H476A) | Lab stock |
| FYJ915 | BL21 carrying pET21a*::mcr-1* | Lab stock |
| FYJ916 | BL21 carrying pET21a*::mcr-2* | Lab stock |
| FYJ917 | BL21 carrying pET21a*::*tm1*-mcr-2* | Lab stock |
| FYJ918 | BL21 carrying pET21a*::*tm2*-mcr-1* | Lab stock |
| FYJ1087 | MG1655 carrying pBAD24*::mcr-2*(N106A) | This work |
| FYJ1088 | MG1655 carrying pBAD24*::mcr-2*(T110A) | This work |
| FYJ1089 | MG1655 carrying pBAD24*::mcr-2*(E114A) | This work |
| FYJ1090 | MG1655 carrying pBAD24*::mcr-2*(S328A) | This work |
| FYJ1091 | MG1655 carrying pBAD24*::mcr-2*(K331A) | This work |
| FYJ1092 | MG1655 carrying pBAD24*::mcr-2*(H388A) | This work |
| FYJ1093 | MG1655 carrying pBAD24*::*tm1-*eptA* | This work |
| FYJ1094 | MG1655 carrying pBAD24*::*tm-*mcr-1* | This work |
| FYJ1095 | MG1655 carrying pBAD24*::*tm2-*eptA* | This work |
| FYJ1096 | MG1655 carrying pBAD24*::*tm-*mcr-2* | This work |
| FYJ1097 | BL21(pLysS) carrying pET21a*::eptA* | This work |
| FYJ1098 | BL21(pLysS) carrying pET21a*::*tm1-*eptA* | This work |
| FYJ1099 | BL21(pLysS) carrying pET21a*::*tm-*mcr-1* | This work |
| FYJ1100 | BL21(pLysS) carrying pET21a*::*tm2-*eptA* | This work |
| FYJ1101 | BL21(pLysS) carrying pET21a*::*tm-*mcr-2* | This work |
| FYJ1102 | MG1655 carrying pBAD24*::eptA*(N106A) | This work |
| FYJ1103 | MG1655 carrying pBAD24*::eptA*(T110A) | This work |
| FYJ1104 | MG1655 carrying pBAD24*::eptA*(E114A) | This work |
| FYJ1105 | MG1655 carrying pBAD24*::eptA*(E240A) | This work |
| FYJ1106 | MG1655 carrying pBAD24*::eptA*(T280A) | This work |
| FYJ1107 | MG1655 carrying pBAD24*::eptA*(S325A) | This work |
| FYJ1108 | MG1655 carrying pBAD24*::eptA*(K328A) | This work |
| FYJ1109 | MG1655 carrying pBAD24*::eptA*(H378A) | This work |
| FYJ1110 | MG1655 carrying pBAD24*::eptA*(H383A) | This work |
| FYJ1111 | MG1655 carrying pBAD24*::eptA*(D452A) | This work |
| FYJ1112 | MG1655 carrying pBAD24*::eptA*(H453A) | This work |
| FYJ1113 | MG1655 carrying pBAD24*::eptA*(H465A) | This work |
| FYJ1114 | BL21(pLysS) carrying pET21a*::mcr-2*(N106A) | This work |
| FYJ1115 | BL21(pLysS) carrying pET21a*::mcr-2*(T110A) | This work |
| FYJ1116 | BL21(pLysS) carrying pET21a*::mcr-2*(E114A) | This work |
| FYJ1117 | BL21(pLysS) carrying pET21a*::mcr-2*(E244A) | This work |
| FYJ1118 | BL21(pLysS) carrying pET21a*::mcr-2*(T283A) | This work |
| FYJ1119 | BL21(pLysS) carrying pET21a*::mcr-2*(S328A) | This work |
| FYJ1120 | BL21(pLysS) carrying pET21a*::mcr-2*(K331A) | This work |
| FYJ1121 | BL21(pLysS) carrying pET21a*::mcr-2*(H383A) | This work |
| FYJ1122 | BL21(pLysS) carrying pET21a*::mcr-2*(H393A) | This work |
| FYJ1123 | BL21(pLysS) carrying pET21a*::mcr-2*(D463A) | This work |
| FYJ1124 | BL21(pLysS) carrying pET21a*::mcr-2*(H464A) | This work |
| FYJ1125 | BL21(pLysS) carrying pET21a*::mcr-2*(H476A) | This work |
| Plasmids |  |  |
| pBAD24 | Arabinose inducible promoter-driven expression vector; Amp^R^ | Lab stock |
| pBAD24*::mcr-1* | A pBAD24 carrying the wild-type version of *mcr-1* at the two cuts of EcoRI and SalI; Amp^R^ | Lab stock |
| pBAD24*::eptA* | A pBAD24 carrying the wild-type version of *eptA* at the two cuts of EcoRI and SalI; Amp^R^ | Lab stock |
| pBAD24*::mcr-2* | A pBAD24 carrying the wild-type version of *mcr-2* at the two cuts of EcoRI and SalI; Amp^R^ | Lab stock |
| pBAD24*::mcr-2*(E244A) | pBAD24 encoding the mutant version of  *mcr-2*(E244A); Amp^R^ | Lab stock |
| pBAD24*::mcr-2*(T283A) | pBAD24 encoding the mutant version of  *mcr-2*(T283A); Amp^R^ | Lab stock |
| pBAD24*::mcr-2*(D463A) | pBAD24 encoding the mutant version of  *mcr-2*(D463A); Amp^R^ | Lab stock |
| pBAD24*::mcr-2*(H464A) | pBAD24 encoding the mutant version of  *mcr-2*(H464A); Amp^R^ | Lab stock |
| pBAD24*::mcr-2*(H393A) | pBAD24 encoding the mutant version of  *mcr-2*(H393A); Amp^R^ | Lab stock |
| pBAD24*::mcr-2*(H476A) | pBAD24 encoding the mutant version of  *mcr-2*(H476A); Amp^R^ | Lab stock |
| pET21a*::mcr-1* | A pET21a carrying the wild-type version of *mcr-1* at the two cuts of NdeI and XhoI; Amp^R^ | Lab stock |
| pET21a*::mcr-2* | A pET21a carrying the wild-type version of *mcr-2* at the two cuts of NdeI and XhoI; Amp^R^ | Lab stock |
| pBAD24*::*tm1-*eptA* | pBAD24 encoding a chimeric MCR version comprising the transmembrane region of *mcr-1* and the extracellular domain of *eptA*; Amp^R^ | Lab stock |
| pBAD24*::*tm-*mcr-1* | pBAD24 encoding a chimeric MCR version comprising the transmembrane region of *eptA* and the extracellular domain of *mcr-1*; Amp^R^ | Lab stock |
| pBAD24*::*tm2-*eptA* | pBAD24 encoding a chimeric MCR version comprising the transmembrane region of *mcr-2* and the extracellular domain of *eptA*; Amp^R^ | This work |
| pBAD24*::*tm-*mcr-2* | pBAD24 encoding a chimeric MCR version comprising the transmembrane region of *eptA* and the extracellular domain of *mcr-2*; Amp^R^ | This work |
| pET21a*::eptA* | A pET21a carrying the wild-type version of *eptA* at the two cuts of NdeI and XhoI; Amp^R^ | This work |
| pET21a*::*tm1-*eptA* | A pET21a carrying a chimeric MCR version comprising the transmembrane region of *mcr-1* and the extracellular domain of *eptA* at the two cuts of NdeI and XhoI; Amp^R^ | This work |
| pET21a*::*tm-*mcr-1* | A pET21a carrying a chimeric MCR version comprising the transmembrane region of *eptA* and the extracellular domain of *mcr-1* at the two cuts of NdeI and XhoI; Amp^R^ | This work |
| pET21a*::*tm2-*eptA* | A pET21a carrying a chimeric MCR version comprising the transmembrane region of *mcr-2* and the extracellular domain of *eptA* at the two cuts of NdeI and XhoI; Amp^R^ | This work |
| pET21a*::*tm-*mcr-2* | A pET21a carrying a chimeric MCR version comprising the transmembrane region of *eptA* and the extracellular domain of *mcr-2* at the two cuts of NdeI and XhoI; Amp^R^ | This work |
| pBAD24*::eptA*(N106A) | pBAD24 encoding the mutant version of  *mcr-1*(N106A); Amp^R^ | This work |
| pBAD24*::eptA* (T110A) | pBAD24 encoding the mutant version of  *mcr-1*(T110A); Amp^R^ | This work |
| pBAD24*::eptA* (E114A) | pBAD24 encoding the mutant version of  *mcr-1*(E114A); Amp^R^ | This work |
| pBAD24*::eptA* (E240A) | pBAD24 encoding the mutant version of  *mcr-1*(E240A); Amp^R^ | This work |
| pBAD24*::eptA* (T280A) | pBAD24 encoding the mutant version of  *mcr-1*(T280A); Amp^R^ | This work |
| pBAD24*::eptA* (S325A) | pBAD24 encoding the mutant version of  *mcr-1*(S325A); Amp^R^ | This work |
| pBAD24*::eptA* (K328A) | pBAD24 encoding the mutant version of  *mcr-1*(K328A); Amp^R^ | This work |
| pBAD24*::eptA* (H378A) | pBAD24 encoding the mutant version of  *mcr-1*(H378A); Amp^R^ | This work |
| pBAD24*::eptA* (H383A) | pBAD24 encoding the mutant version of  *mcr-1*(H383A); Amp^R^ | This work |
| pBAD24*::eptA* (D452A) | pBAD24 encoding the mutant version of  *mcr-1*(D452A); Amp^R^ | This work |
| pBAD24*::eptA* (H453A) | pBAD24 encoding the mutant version of  *mcr-1*(H453A); Amp^R^ | This work |
| pBAD24*::eptA* (H465A) | pBAD24 encoding the mutant version of  *mcr-1*(H465A); Amp^R^ | This work |
| pET21a*::mcr-2*(N106A) | A pET21a carrying the wild-type version of *mcr-2*(N106A) at the two cuts of NdeI and XhoI; Amp^R^ | This work |
| pET21a*::mcr-2*(T110A) | A pET21a carrying the wild-type version of *mcr-2*(T110A) at the two cuts of NdeI and XhoI; Amp^R^ | This work |
| pET21a*::mcr-2*(E114A) | A pET21a carrying the wild-type version of *mcr-2*(E114A) at the two cuts of NdeI and XhoI; Amp^R^ | This work |
| pET21a*::mcr-2*(E244A) | A pET21a carrying the wild-type version of *mcr-2*(E244A) at the two cuts of NdeI and XhoI; Amp^R^ | This work |
| pET21a*::mcr-2*(T283A) | A pET21a carrying the wild-type version of *mcr-2*(T283A) at the two cuts of NdeI and XhoI; Amp^R^ | This work |
| pET21a*::mcr-2*(S328A) | A pET21a carrying the wild-type version of *mcr-2*(S328A) at the two cuts of NdeI and XhoI; Amp^R^ | This work |
| pET21a*::mcr-2*(K331A) | A pET21a carrying the wild-type version of *mcr-2*(K331A) at the two cuts of NdeI and XhoI; Amp^R^ | This work |
| pET21a*::mcr-2*(H383A) | A pET21a carrying the wild-type version of *mcr-2*(H383A) at the two cuts of NdeI and XhoI; Amp^R^ | This work |
| pET21a*::mcr-2*(H393A) | A pET21a carrying the wild-type version of *mcr-2*(H393A) at the two cuts of NdeI and XhoI; Amp^R^ | This work |
| pET21a*::mcr-2*(D463A) | A pET21a carrying the wild-type version of *mcr-2*(D463A) at the two cuts of NdeI and XhoI; Amp^R^ | This work |
| pET21a*::mcr-2*(H464A) | A pET21a carrying the wild-type version of *mcr-2*(H464A) at the two cuts of NdeI and XhoI; Amp^R^ | This work |
| pET21a*::mcr-2*(H476A) | A pET21a carrying the wild-type version of *mcr-2*(H476A) at the two cuts of NdeI and XhoI; Amp^R^ | This work |
