# Supplementary material for: An Evolutionarily Conserved Mechanism for Intrinsic and Transferable Polymyxin Resistance
Source: mBio. 2018 Apr 10;9(2):e02317-17. doi: 10.1128/mBio.02317-17 (PMC5893884; doi:10.1128/mBio.02317-17)
Supplement: TABLE S2 [file mbo002183817st2.doc]

**Table S2** Primers used in this study

| **Primers** | **Sequences** |
| --- | --- |
| MCR-2(N106A)-F | 5’-ATG CTC CAA GCA GCC ATG CAA ACC GAC CAA GC-3’ |
| MCR-2(N106A)-R | 5’-ATG GCT GCT TGG AGC ATG GTG GTG TCA TAG AC-3’ |
| MCR-2(T110A)-F | 5’-CAT GCA AGC AGA CCA AGC CGA GTC TAA GGA CTT-3’ |
| MCR-2(T110A)-R | 5’-CTT GGT CTG CTT GCA TGG CAT TTT GGA GCA TG-3’ |
| MCR-2(E114A)-F | 5’-AAG CCG CAA CCA AGG ATC TAT TAA ACG CAG CG-3’ |
| MCR-2(E114A)-R | 5’-CTT GGT TGC GGC TTG GTC GGT CTG TAG GG -3’ |
| MCR-2(S328A)-F | 5’-ATG CAG ACT CAA AAG GCG TGA TGG ATA AGC TA-3’ |
| MCR-2(S328A)-R | 5’-GCC TTT TGA GTC TGC ATT ATT ATC ACG CCA CAA GAT ACC-3’ |
| MCR-2(K331A)-F | 5’-ATT CAG ACT CAG CAG GCG TGA TGG ATA AGC TAC CTG-3’ |
| MCR-2(K331A)-R | 5’-CTG CTG AGT CTG AAT TAT TAT CAC GCC ACA-3’ |
| MCR-2(H388A)-F | 5’-CAT GCT AGC ACA AAT GGG CAA TCA TGG GCC GG-3’ |
| MCR-2(H388A)-R | 5’-CCA TTT GTG CTA GCA TGA TGA GCA TAT CTT TGC C-3’ |
| MCR-1-F(NdeI) | 5’-GGAATTC CATATG ATG CAG CAT ACT TCT GTG TG-3’ |
| MCR-1-R(XhoI) | 5’-CCG CTCGAG GCG GAT GAA TGC GGT GC-3’ |
| MCR-2-F(NdeI) | 5’-G GAA TTC CATATG ACA TCA CAT CAC TCT TGG TAT-3’ |
| MCR-2-R(XhoI) | 5’-CCG CTCGAG CTG GAT AAA TGC CGC GCG-3’ |
| EptA-F(NdeI) | 5’-GGAA TTC CATATG ATA AAA CCG AAC CTG AGG CCG A-3’ |
| EptA-R (XhoI) | 5’-CCG CTCGAG GCG CGG ACG GCG GCA GGC TGC CAA TAT-3’ |
| MCR-1-F (EcoRI) | 5’-CG GAATTC ATGATG CAG CAT ACT TCT GTGT-3’ |
| MCR-1-TM-R | 5’-TTG CGG AAA AAC GAG GCG TAA TGA CTG CTG AAC GCC ACC-3’ |
| MCR-1-OS-F | 5’-CAA TGT TGC AAT ATC AGG ATT ATG CCA GTT TCT TTC GCG-3’ |
| MCR-1-R (SalI) | 5’-CCG GTCGAC TCA GCG GAT GAA TGC GGT-3’ |
| MCR-2-F (EcoRI) | 5’-CG GAATTC ATG ACA TCA CAT CAC TCT TG-3’ |
| MCR-2-TM-R | 5’-CTG ACT GCT AAA TAG TCC AAT CGG C-3’ |
| MCR-2-OS-F | 5’-ATC GCA ATG TTG CAA TAT CAG GAT TAT GCG AGT TTC TTT CGG GTG-3’ |
| MCR-2-R (SalI) | 5’-CCG GTCGAC TTA CTG GAT AAA TGC CGC GC-3’ |
| EptA-F (EcoRI) | 5’-AACC GAATTC ATG ATA AAA CCG AAC CTG AGG CC-3’ |
| EptA-TM-R | 5’-CGC GAA AGA AAC TGG CAT AAT CCT GAT ATT GCA ACA TTG-3’ |
| EptA-OS-F | 5’-GGT GGC GTT CAG CAG TCA TTA CGC CTC GTT TTT CCG CAA-3’ |
| EptA-R (SalI) | 5’-CCG GTCGAC TCA GCG CGG ACG GCG GCA G-3’ |
| EptA(N106A)-F | 5’-GTTGAA TGC AGT CTT GCA AAC CAC GGC GGC GG-3’ |
| EptA(N106A)-R | 5’-GCA AGA CTG CAT TCA ACA TCG ACT TGT TGA AAT AGA T-3’ |
| EptA(T110A)-F | 5’-CTT GCA AGC AAC GGC GGC GGA AAG CGC GC-3’ |
| EptA(T110A)-R | 5’-CGT TGC TTG CAA GAC ATT ATT CAA CAT CGA C-3’ |
| EptA(E114A)-F | 5’-AAC CAC GGC GGC GGC AAG CGC GCG CCT GAT TAC G-3’ |
| EptA(E114A)-R | 5’-TTG CCG CCG CCG TGG TTT GCA AGA CAT TAT TC-3’ |
| EptA(E240A)-F | 5’-TCG TGG GCG CAA CTA CGC GTG CCG CCA ACT GG -3’ |
| EptA(E240A)-R | 5’-CGT AGT TGC GCC CAC GAC CAG CAC CAC GAA AC-3’ |
| EptA(T280A)-F | 5’-CAC ATC GGC AGC GCA CTC CCT GCC GTG TAT GT-3’ |
| EptA(T280A)-R | 5’-AGT GCG CTG CCG ATG TGC CGC AGC TTC TGA CC-3’ |
| EptA(S325A)-F | 5’-AAA CGA TGC AGG CTG CAA GGG CGT GTG CGG CA-3’ |
| EptA(S325A)-R | 5’-TGC AGC CTG CAT CGT TTT CCA ACC AAG TAA CTT CC-3’ |
| EptA(K328A)-F | 5’-ACG ATT CCG GCT GCG CAG GCG TGT GCG GCA AAG TG-3’ |
| EptA(K328A)-R | 5’-TGC GCA GCC GGA ATC GTT TTC CAA CCA AGT AA-3’ |
| EptA(H378A)-F | 5’-AAT CCT GGC AAC CAT CGG CAG CCA CGG GCC GA-3’ |
| EptA(H378A)-R | 5’-CGA TGG TTG CCA GGA TTA AAA CCG CGT CTT TAT-3’ |
| EptA(H383A)-F | 5’-ATA CCA TCG GCA GCG CAG GGC CGA CGT ATT ACG AAC G-3’ |
| EptA(H383A)-R | 5’-TGC GCT GCC GAT GGT ATG CAG GAT TAA AAC CG-3’ |
| EptA(D452A)-F | 5’-TAT GTT TCC GCA CAC GGC GAA AGT TTG GGC GA-3’ |
| EptA(D452A)-R | 5’-CCG TGT GCG GAA ACA TAA TAC ACC GAG CTT TCC-3’ |
| EptA(H453A)-F | 5’-GAC GCA GGC GAA AGT TTG GGC GAA AAC GGG AT-3’ |
| EptA(H453A)-R | 5’-AAA CTT TCG CCT GCG TCG GAA ACA TAA TAC ACC GAG C-3’ |
| EptA(H465A)-F | 5’-ACG GGA TGT ACC TGG CAG CCG CGC CTT ACG CCA TC-3’ |
| EptA(H465A)-R | 5’-TGC CAG GTA CAT CCC GTT TTC GCC CAA ACT TT-3’ |

*The underlined letters in italic denote restrictions sites.
